# NSUN2-mediated m^5^C modification drives alternative splicing reprogramming and promotes multidrug resistance in anaplastic thyroid cancer through the NSUN2/SRSF6/UAP1 signaling axis

**DOI:** 10.7150/thno.104713

**Published:** 2025-01-27

**Authors:** Xiukun Hou, Qiman Dong, Jie Hao, Min Liu, Junya Ning, Mei Tao, Zhongyu Wang, Fengli Guo, Dongmei Huang, Xianle Shi, Ming Gao, Dapeng Li, Xiangqian Zheng

**Affiliations:** 1 Department of Thyroid and Neck Cancer, Tianjin Medical University Cancer Institute and Hospital, National Clinical Research Center for Cancer, Key Laboratory of Cancer Prevention and Therapy, Tianjin's Clinical Research Center for Cancer, Tianjin 300040, China.; 2 Department of Thyroid and Breast Surgery, Tianjin Union Medical Center, Tianjin 300121, China.; 3 Tianjin Key Laboratory of General Surgery in Construction, Tianjin Union Medical Center, Tianjin 300121, China.; 4 School of Medicine, Nankai University, Tianjin 300071, China.; 5 Center for Stem Cell Biology and Regenerative Medicine, MOE Key Laboratory of Bioinformatics, School of Life Sciences, Tsinghua University, Beijing 100084, China.

**Keywords:** 5-methylcytosine, anaplastic thyroid cancer, multidrug resistance, NSUN2, alternative splicing reprogramming

## Abstract

**Rationale:** Anaplastic thyroid carcinoma (ATC) is an extraordinarily aggressive form of thyroid cancer, frequently presenting with locally advanced infiltration or distant metastases at the time of initial diagnosis, thus missing the optimal window for surgical intervention. Consequently, systemic chemotherapy and targeted therapies are vital for improving the prognosis of ATC. However, ATC exhibits significant resistance to conventional treatments, highlighting the need to elucidate the biological mechanisms underlying this drug resistance and identify novel therapeutic targets to overcome it.

**Methods:** We conducted a comprehensive analysis of both bulk and single-cell RNA sequencing (scRNA-seq) data from ATC samples to screen for m^5^C modification-related genes associated with multidrug resistance (MDR). We then performed IC_50_ assays, flow cytometry, and employed a spontaneous tumorigenic ATC mouse model with Nsun2 knockout to demonstrate that NSUN2 promotes MDR in ATC. To investigate the mechanisms of NSUN2-mediated drug resistance, we generated NSUN2-knockout ATC cell lines and performed transcriptomic, proteomic, and MeRIP-seq analyses. Additionally, RNA sequencing and alternative splicing analyses were conducted to determine global changes upon NSUN2 knockout. We further explored the underlying mechanisms of the NSUN2/SRSF6/UAP1 axis through glycoprotein staining, denaturing IP ubiquitination, nuclear-cytoplasmic fractionation, and PCR. Lastly, we evaluated the synergistic effects of a small-molecule NSUN2 inhibitor with anticancer agents both *in vitro* and *in vivo*.

**Results:** Our findings reveal that NSUN2 expression correlates significantly with MDR in ATC. NSUN2 operates as a "writer" and ALYREF as a "reader" of m^5^C on SRSF6 mRNA, inducing alternative splicing reprogramming and redirecting the splice form of the UAP1 gene from AGX1 to AGX2. As a result, AGX2 enhances the N-linked glycosylation of ABC transporters, stabilizing them by preventing ubiquitination-mediated degradation. Furthermore, an NSUN2 inhibitor reduces NSUN2 enzymatic activity and diminishes downstream target expression, presenting a novel, promising therapeutic approach to overcome MDR in ATC.

**Conclusions:** These findings suggest that the NSUN2/SRSF6/UAP1 signaling axis plays a vital role in MDR of ATC and identify NSUN2 as a synergistic target for chemotherapy and targeted therapy in ATC.

## Introduction

Anaplastic thyroid carcinoma (ATC) is among the most aggressive malignancies and is consistently associated with poor prognosis. Although it accounts for only 1-2% of all thyroid cancers, it contributes to 14% of thyroid cancer-related mortalities 1. The majority of ATC patients present with locally advanced infiltration or distant metastases at initial diagnosis, thereby missing the optimal surgical window. Consequently, systemic chemotherapy and targeted therapies are critical for improving ATC outcomes. However, the high expression of ABC transporters in ATC actively expels multiple drugs from cells, resulting in multidrug resistance (MDR) 2. The molecular mechanisms governing this elevated ABC transporter expression remain largely unknown, underscoring the need to elucidate mechanisms of drug resistance and identify novel therapeutic targets for ATC.

RNA modifications, particularly N6-methyladenosine (m^6^A), have been widely implicated in tumorigenesis and tumor progression 3. Notably, 5-methylcytosine (m^5^C), another prevalent RNA modification, has been implicated in multiple tumorigenic processes by regulating RNA stability, nucleocytoplasmic shuttling, and splicing 4-6. Splicing is the process by which introns are removed from pre-mRNAs, and the remaining exons are ligated to form mature mRNA. During this process, different combinations of exons produce distinct mature mRNAs, a phenomenon known as alternative splicing (AS). Recently, an increasing number of studies have revealed that AS is intricately linked to various cancer-related physiological processes, including drug resistance 7-9. Malignant cells can modulate AS to evade therapeutic interventions by altering the splicing patterns of genes involved in proliferation, apoptosis, and drug metabolism 10-12. Nevertheless, the role and molecular mechanisms of RNA m^5^C modification-mediated AS in promoting drug resistance remain largely unexplored.

N-linked glycosylation is one of the most common post-translational modifications of membrane-bound proteins, influencing numerous tumorigenic processes 13, 14. Growing evidence indicates that glycosylation of cell membrane proteins is critical for maintaining protein stability, highlighting glycosylation as a potential therapeutic target in combination cancer therapies 15-17. The ATP-binding cassette (ABC) transporters form a family of proteins that mediate multidrug resistance 18, 19. Numerous studies have been conducted to elucidate the role of ABC transporters in ATC multidrug resistance 20. Among these, ABCG2 and ABCC1 are the most highly expressed transporters in ATC and have been identified as playing pivotal roles in MDR 2, 21. Furthermore, accumulating studies have demonstrated that defects in N-linked glycosylation of ABC transporters can regulate their protein levels 22, 23. Therefore, glycosylation is considered a critical regulatory mechanism of ABC transporter expression, contributing to MDR. However, the molecular mechanisms linking N-linked glycosylation and ABC transporters in ATC remain largely unexplored.

Here, our study demonstrates the correlation between NSUN2 expression and MDR in ATC and elucidate the underlying genetic and molecular mechanisms. Specifically, NSUN2 drives alternative splicing by facilitating the nucleocytoplasmic shuttling of SRSF6 in an m^5^C-dependent manner, resulting in enhanced N-linked glycosylation in ATC cells. The elevated glycosylation of ABC transporters subsequently increases their stability and promotes MDR in ATC. Moreover, an NSUN2 inhibitor diminishes its enzymatic function and the expression of key downstream targets, suggesting a promising strategy to combat MDR in ATC. Taken together, our findings reveal that the NSUN2/SRSF6/UAP1 axis promotes MDR in ATC and establishes a link between N-linked glycosylation and MDR.

## Methods

### Drug sensitivity analysis

The R package “oncoPredict” was used to calculate the IC_50_ Area Under the Curve (AUC) values of potential drug responses 24. Training data of drugs and drug responses were obtained from the Cancer Therapeutics Response Portal (CTRP). An absolute correlation coefficient > 0.3 and P-value <0.05 were used as the thresholds for significance.

### Processing of scRNA-seq data

Processed single-cell data and primary cell annotation information were downloaded from GEO to further identify ATC tumor-derived cells based on the origin of malignant cells 25. Then, cells with NSUN2 expression of 0 were considered NSUN2-negative cells, and cells with NSUN2 expression greater than 0 were considered NSUN2-positive cells.

### Differential drug sensitivity analysis

In the malignant ATC scRNA-seq dataset, NSUN2-negative and NSUN2-positive cells were randomly grouped into 50 subsets, and each subset's mean expression values were used as pseudo-bulk samples. We utilized the calcPhenotype function to calculate the IC_50_ AUC values for each drug in the CTRP database. The “DESeq2” package was employed to assess differential drug sensitivity between NSUN2-positive and NSUN2-negative cells, while the “ggplot2” package was used to generate a volcano plot of the differential drug responses. Drugs were considered significant if P < 0.05 and log2FC > 0.

### Gene set functional analysis

Gene Ontology (GO) gene sets were obtained from the R package “msigdbr,” and functional analyses of these gene sets were performed using the “GSVA” package 26, 27. The correlation between GO pathways and NSUN2 gene expression was calculated using the Spearman method. The “linkET” package was employed to generate a heatmap illustrating the correlations between drugs and NSUN2-associated pathways.

### Single-cell drug susceptibility evaluation

The R package “beyondcell” was utilized to assess drug sensitivity in scRNA-seq data, with parameter settings as described in a previous study 28, 29. The Spearman method was then employed to analyze the correlation between the Beyondcell score and NSUN2 expression in each ATC cell.

### Data availability

The datasets supporting the analysis of drug sensitivity comprise 52 ATC samples obtained from multiple publicly available Gene Expression Omnibus (GEO) datasets, including GSE29265, GSE33630, GSE65144, and GSE76039. Additionally, single-cell data were sourced from GSE193581. All referenced data are available in the GEO repository, which can be accessed at https://www.ncbi.nlm.nih.gov/geo/.

### Cell culture

Two anaplastic thyroid carcinoma cell lines (Cal-62 and ACT-1) were used in this study. The cell lines were purchased from the Chinese Academy of Sciences (Shanghai, China). All the cell lines were cultured in Roswell Park Memorial Institute Medium (RPMI)-1640 medium or Dulbecco's Modified Eagle Medium (DMEM) (Gibco, USA) supplemented with 10% fetal bovine serum (FBS) and 1% penicillin and streptomycin and maintained in a humidified atmosphere with 5% CO2 at 37 °C.

### Vectors, RNA interference

Human shRNAs were expressed using the pLKO.1 vector. ABCC1 mutants [N71Q, N19Q, N310Q, N(71+310)Q] and ABCG2 mutants (N557Q, N338Q, N596Q) were generated through site-directed mutagenesis using the pCDH-ABCC1/ABCG2 vectors as a template. Additionally, pLVX-NSUN2, pLVX-NSUN2 C271A, pCDH-ALYREF, and pCDH-ALYREF K171A, pCDH-SRSF6 were obtained from HANBIO, China. All primers used for cloning and siRNA sequences are detailed in Table S1. Lipofectamine 2000 (Invitrogen, USA) was employed to transfect siRNA or plasmids, following the manufacturer's instructions. HEK293T cells were utilized to generate lentiviral particles. Lentiviral particles were collected after 48 and 72 hours, filtered through a 0.45 μm filter, and concentrated using Amicon Ultra-15 Centrifugal Filters (Merck).

### Western blotting

Proteins were extracted from cells using radioimmunoprecipitation assay (RIPA) buffer (Solarbio, Beijing, China) following the manufacturer's protocol. Protein concentrations were quantified using the BCA assay. Samples were resolved on 8-12% sodium dodecyl sulfate-polyacrylamide gel electrophoresis (SDS-PAGE) gels and transferred onto polyvinylidene difluoride (PVDF) membranes. Subsequently, the membranes were incubated with primary antibodies followed by peroxidase-conjugated anti-mouse or anti-rabbit IgG secondary antibodies. Finally, the membranes were visualized using ECL Plus reagents (Cell Signaling Technology, Danvers, MA, USA). The primary and secondary antibodies used in this study are detailed in Table S2.

### Immunohistochemistry (IHC) staining assay

The tissue samples of the tumors were subjected to immunohistochemical staining according to a standard protocol. The signal was visualized with the DAB Substrate Kit (MaiXin Bio, China). The staining scores were analyzed from three independent, random fields and were evaluated by two experienced pathologists. The staining intensity was graded as 0 (no staining), 1 (slightly brown), 2 (moderately brown) and 3 (dark brown). The percentage of positive cells was scored as 0 (<25%), 1 (25-50%), 2 (50-75%), and 3 (>75%). The total staining score for each sample was calculated as the sum of the staining intensity and percentage of positively stained cells. Samples with final score ≥4 were classified as high expression, and those with final score <4 as low expression. The Ethics Committee of Tianjin Medical University Cancer Hospital approved this study (Approval ID: Ek2021149). Informed consent was acquired to conduct the experiments using human tissues.

### Cell viability

A total of 1000-1500 cells per well were seeded into 96-well plates. Cell lines were exposed to the drugs or DMSO for 48h. Subsequently, cell viability was assessed using the Cell Counting Kit-8 (CCK-8) following the manufacturer's protocol. All IC_50_ values represent averages of replicates relative to cell viability n DMSO-treated cells, normalized to 100%. The small-molecule compounds or drugs used in this study are detailed in Table S3.

### Dot blot

Total RNA was extracted using TRIzol reagent (Invitrogen, Carlsbad, USA) and subsequently treated with deoxyribonuclease I (DNase). Enrichment of mRNA from total RNA was performed using Dynabeads™ mRNA Purification Kit (Invitrogen, Carlsbad, USA). Briefly, Superparamagnetic Dynabeads™ coupled with oligo-(dT)25 were equilibrated in Binding Buffer and subsequently mixed with purified total RNA. Following the binding step, the beads were washed to eliminate contaminating RNA species. mRNA was then eluted with 10 mM Tris-HCl. Different amounts of mRNA were then loaded onto an Amersham Hybond N+ membrane. After ultraviolet crosslinking for 5 minutes at 254 nm, the membrane was blocked with non-fat milk in 1X TBST for 1 hour at room temperature, followed by incubation with an anti-m5C antibody and the corresponding secondary antibody. Subsequently, the membrane was washed with TBST and visualized using ECL Plus reagents (Cell Signaling Technology, Danvers, MA, USA). Finally, the membrane was washed and stained with methylene blue.

### M^5^C quantification assay

The change of global m^5^C levels in mRNA was measured by EpiQuik m^5^C RNA Methylation Quantification Kit (Colorimetric) (Epigentek) following the manufacturer's protocol. In summary, a negative control and a standard curve with six varying concentrations (ranging from 0.02 to 1 ng of m^5^C) were established. Each assay included 200 ng of mRNA. Following the binding of mRNA to the 96-well plates, the binding solution was discarded, and the plates were washed three times with a diluted wash buffer. Subsequently, diluted capture anti- m^5^C antibodies were introduced, and the plates underwent four additional washes with the diluted wash buffer. Afterward, 100 μl of developer solution was added to each well and incubated at room temperature for 7 minutes in the dark. The enzyme reactions were terminated by adding 100 μl of stop solution to each well. The optical density (OD) was measured at 450 nm using a microplate reader to quantify the absolute amount of m^5^C.

### RNA-seq and differential gene expression analysis

Total RNA was extracted using TRIzol reagent (Invitrogen, USA) following the manufacturer's instructions. The RNA-Seq transcriptome library was prepared using the Illumina® Stranded mRNA Prep, Ligation Kit (Illumina, San Diego, CA). The RNA-Seq library was sequenced using the NovaSeq 6000 sequencer.

To identify differentially expressed genes (DEGs) between samples, the expression level of each transcript was calculated based on fragments per kilobase of transcript per million mapped reads (FPKM). RSEM was employed to quantify gene abundances, and differential expression analysis was conducted using DESeq2. Sequencing data have been deposited in the Gene Expression Omnibus under accession number GSE244824.

### TMT labeling and LC-MS/MS analysis

In brief, total proteins were extracted using a urea lysis buffer containing 7 M urea, 2 M thiourea, and 1% SDS, supplemented with a protease inhibitor. The peptide mixture was labeled using the 10-plex TMT reagent (Thermo Fisher) following the manufacturer's instructions. Labeled peptides were analyzed through online nanoflow liquid chromatography-tandem mass spectrometry (LC-MS/MS). The experiments were conducted on a 9RKFSG2_NCS-3500R system (Thermo, USA) connected to a Q Exactive Plus quadrupole orbitrap mass spectrometer (Thermo, USA).

### Glycoprotein staining

The experiment was performed using a Thermo Scientific Pierce Glycoprotein Staining Kit following the manufacturer's instructions. Briefly, protein samples underwent electrophoresis, and the gel was subsequently fixed with 50% methanol for 30 minutes. The gel was then transferred to 25 mL of Oxidizing Solution for 15 minutes. Subsequently, the gel was placed in Glycoprotein Stain for 15 minutes, followed by transfer to Reducing Solution for 5 minutes. Glycoproteins appeared as magenta bands. Additionally, Coomassie protein staining was performed to detect total protein.

### Nuclear and cytoplasmic RNA extraction

Nuclear and cytoplasmic RNAs were isolated from cells using the Cytoplasmic and Nuclear RNA Purification Kit (Norgen Biotek, Thorold, Canada), following the manufacturer's instructions. GAPDH (control for cytoplasmic transcript) and U6 (control for nuclear transcript) in the cytoplasmic and nuclear fractions were quantified using qRT-PCR analysis. Primer sequences are provided in Table S4.

### M^5^C-RNA immunoprecipitation sequencing (MeRIP-seq) and data analysis

Methylation-RNA immunoprecipitation (MeRIP) was conducted as follows: Initially, isolated RNA underwent random fragmentation via a 3-minute incubation at 75°C in a 1X fragmentation buffer (10 mM Tris-HCl at pH 7, 10 mM ZnCl2). To halt the fragmentation process, a 1X Stop Solution containing 0.05 M EDTA was added. Next, 10 μL of anti-m5C antibody (Active Motif #39649) was incubated with 30 μL of protein G Sepharose (GE Healthcare) in 300 μL of IP buffer (10 mM Tris-HCl at pH 7.5, 150 mM NaCl, 0.05% Triton-X). Following incubation, bead-antibody complexes were washed three times with IP buffer and subsequently adjusted to a volume of 250 μL in IP buffer. A 10 μg sample of RNA was then introduced to the bead-antibody complexes and incubated overnight at 4°C on a rotating wheel. After multiple washes with IP buffer, RNA was exposed to 300 μL of elution buffer (5 mM Tris-HCl at pH 7.5, 1 mM EDTA, 0.05% SDS, 80 μg Proteinase K) for 1 hour at 50°C. The beads were then removed via centrifugation and the supernatant was combined with 800 μL TRIzol (Sigma-Aldrich) to facilitate RNA isolation, as previously described. To aid in the final precipitation, 1 μL of glycogen (20 μg/μL) was used as a carrier. Libraries were constructed and sequenced on the Illumina HiSeq system following a polyadenylated RNA selection protocol per the manufacturer's instructions.

For MeRIP-seq data analysis, after an initial quality check, the raw sequence reads were aligned to the human reference genome (hg19) using TopHat v2.1.1. Subsequently, gene expression levels were quantified using Cufflinks v2.2.1. Normalization of gene expression values was based on the FPKM (Fragments Per Kilobase of transcript per Million mapped reads) method.

### RNA-pull down assay

The procedure was adapted from a previous report 30. Briefly, Cell lysates were prepared with lysis buffer (150 mM KCl, 10 mM HEPES pH 7.6, 2 mM EDTA, 0.5% NP-40, 0.5 mM DTT, 1:100 protease inhibitor cocktail, 400 U/ml RNase inhibitor), and incubated at 4 °C for 30 min with rotation. Then, mix 50 µl streptavidin magnetic beads with 3′-biotin-labelled RNA probes and incubated for 30 min with agitation. After incubation, add 100 mg protein lysate and incubated for 60 min at 4°C with agitation. The RNA-protein-bead mixture was heated at 95 °C for 5 min.

### RIP-qPCR

M^5^C-modified transcripts were immunoprecipitated as previously described. Briefly, fragmented mRNA was captured using Anti-Immunoglobulin G (Anti-IgG) or Anti-m5C antibody. Next, the RNA in the immunoprecipitates was isolated and subjected to qRT-PCR as described below. Primers for RT-qPCR are detailed in Table S5.

### Reverse transcription followed by PCR (RT-PCR) and quantitative PCR (RT-qPCR)

Total RNA was extracted using TRIzol reagent (Invitrogen, USA). 1μg of total RNA was reverse transcribed into cDNA using the HiScript III RT SuperMix qPCR (+gDNA wiper) (Vazyme). Subsequently, PCR was performed using 2X Rapid Taq Master Mix (Vazyme) for RT-PCRs. Quantitative PCR (qPCR) was executed using 2X HQ SYBR PCR Mix (Zomanbio). β-Actin and GAPDH served as loading controls for RT-PCRs and RT-qPCRs. Primers are described in Table S6 and S7.

### Flow cytometry

Intracellular accumulation of doxorubicin, cisplatin, and lenvatinib was analyzed by flow cytometry following treatment at a fixed concentration. Briefly, cells were plated in 6-well plates at 2×10^5^ cells per well and treated 24 hours later with doxorubicin, cisplatin, and lenvatinib. We incubated the tumor cells with drug-containing medium for 6 hours. After the initial incubation, the drug-containing medium was replaced with drug-free medium, and the cells were incubated for an additional 6 hours. We then collected the cells, washed twice with 1× PBS, and resuspended in 500 μL of 1× PBS. Fluorescence associated with the cells was then measured and calculated as mean fluorescence intensity (MFI) for each sample. Cisplatin-CY5 and lenvatinib-FITC were purchased from Xi'an Ruixi Biological Technology. Analysis was carried out in triplicate for at least three separate experiments.

### Animal studies

We developed a spontaneous tumorigenic model of murine anaplastic thyroid carcinoma (mATC) with an Nsun2 knockout by crossing Nsun2^flox/flox^ mice with TPO-cre;Braf^V600E^;Trp53^ flox/flox^ mice.

NSUN2^flox/flox^ mice were purchased from Cyagen Biosciences (Shuzhou). Tumor induction was achieved through intraperitoneal (i.p.) administration of tamoxifen at a dose of 150 mg/kg, dissolved in corn oil, with two administrations at 8 weeks of age. The mice were then subjected to intraperitoneal injections of 5 mg/kg doxorubicin or cisplatin once per week, or oral administration of lenvatinib at 10 mg/kg, once every three days. The NSUN2 inhibitor (50mg/kg) was administered via intraperitoneal injection twice every six days.

To establish a xenograft tumor model, suspensions of 1 × 10^7^ Cal-62 cells treated with indicated vectors were injected into 5-week-old female NCG mice (each group, n = 5). Two weeks later, the mice received intraperitoneal injections of doxorubicin or cisplatin at 5 mg/kg once per week, or oral administration of lenvatinib at 10 mg/kg once every three days. Tumors were measured every three days. All mice were purchased from SPF Biotechnology (Beijing, China).

All animal studies were approved by the Ethics Committee of the Tianjin Medical University Cancer Institute and Hospital and were conducted in accordance with the National Institutes of Health Guide for the Care and Use of Laboratory Animals (Approved No: NSFC-AE-2023162).

### Statistics

All data in this manuscript were collected from three independent experiments. Data are presented as the mean ± SD. Kaplan-Meier analysis was performed to evaluate survival curves. We performed the statistical analysis using GraphPad Prism 9.0 software. All data were analyzed with Student's t test (***p < 0.001, **p < 0.01, *p < 0.05).

## Results

### Bioinformatics analysis reveals a correlation between NSUN2 and MDR in ATC

**Figure 1:**
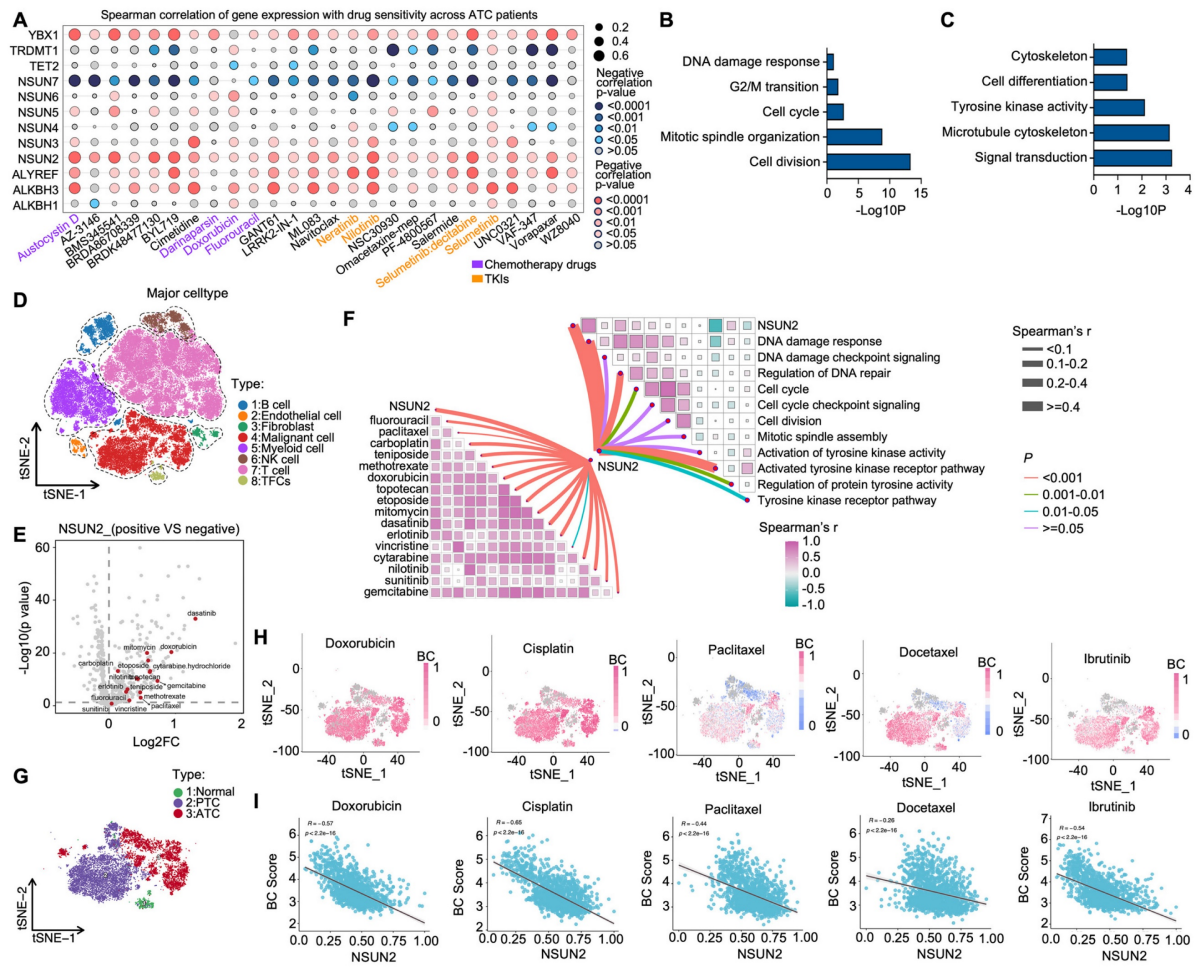
** Bioinformatics analysis reveals a correlation between NSUN2 and MDR in ATC.** A) Spearman correlation of gene expression and drug sensitivity in ATC patients from the CTRP database. B-C) Summary of enriched pathways among differentially expressed genes (DEGs) between high- and low-NSUN2-expression groups. D) TSNE plot exhibiting the identified clusters of the scRNA-seq data from ATC (n = 10). E) Scatterplot depicting the log2 fold change (log2FC) for NSUN2-positive versus NSUN2-negative cells and its correlation with predicted IC_50_ Area Under the Curve (AUC) values for each drug. F) Butterfly diagram illustrating correlations among NSUN2 expression, drug sensitivity, and GO pathway analysis. G) TSNE plot displaying the identified clusters in scRNA-seq data from ATC (n = 10). H) Single-cell drug sensitivity analysis using Beyondcell on GEO dataset samples. I) Correlation between NSUN2 expression and the Beyondcell score.

5-methylcytosine (m^5^C) is a crucial post-transcriptional modification and has been reported to play vital roles in tumor drug resistance 31. To evaluate its function in ATC drug resistance, we examined correlations between the expression of 12 m^5^C regulators [NSUN2-7, TRDMT1 (writers); ALKBH1, TET2, ALKBH3 (erasers); YBX1 and ALYREF (readers)] and drug sensitivity in the Cancer Therapeutics Response Portal (CTRP). We found that NSUN2 expression was positively associated with the IC_50_ values of multiple anticancer agents, including both chemotherapy drugs and tyrosine kinase inhibitors (TKIs) (Figure 1A). Next, using ATC transcriptional data from the GEO database (GSE33630 and GSE76039), we stratified patients into high- and low-NSUN2 expression groups for differential gene expression analysis. Gene Ontology (GO) analysis showed that NSUN2 expression was linked to cell division, mitotic spindle organization, cell cycle, G2/M transition, and DNA damage response—processes frequently targeted by chemotherapy (Figure 1B). Additionally, NSUN2 expression correlated with tyrosine kinase activity, a process typically affected by TKIs (Figure 1C). Further gene set enrichment analysis (GSEA) indicated that higher NSUN2 expression was positively correlated with resistance to both TKIs and chemotherapy drugs (Figure S1A-D), as well as with disruptions in ABC transporter pathways, which mediate multidrug resistance (Figure S1E-F).

To further explore NSUN2's role in ATC drug resistance, we analyzed single-cell RNA-seq data from 10 ATC tissues in the GEO database. Cells were categorized into eight clusters, including malignant cells, B cells, endothelial cells, fibroblasts, and T cells (Figure 1D). Using the Cancer Cell Line Encyclopedia (CCLE) dataset, which comprises 545 drugs and 829 cell lines with IC_50_ AUC data and gene expression profiles, we observed that NSUN2 expression in ATC cells was positively correlated with the IC_50_ values of various chemotherapy drugs and TKIs (Figure 1E). GO analysis of the single-cell data further linked high NSUN2 expression in ATC cells to pathways involved in DNA damage response, cell division, and tyrosine kinase activity (Figure 1F). To clarify susceptibility of chemotherapy drugs and TKIs in separate malignant cells, three clusters of thyroid carcinoma cells were identified (Figure 1G). Next, the tSNE embeddings were computed using drug-induced transcriptional changes derived from Beyondcell's databases, and clusters were presented according to malignant cells and normal epithelial cells, which is similar to clusters based on gene expression in Figure 1G. As it shown, low Beyondcell scores of the signatures of chemotherapy drugs or TKIs were mainly mapped to ATC cells, which predict ATC cells are resistant with the indicated drugs (Figure 1H). Moreover, in ATC cells, we observed a negative correlation between Beyondcell scores and NSUN2 expression levels. This suggests that higher NSUN2 expression is positively associated with the indicated drug resistance in ATC (Figure 1I). In summary, these results above demonstrated that heightened NSUN2 expression may drive multidrug resistance in ATC.

### NSUN2-induced MDR in ATC depends on its methyltransferase activity

**Figure 2:**
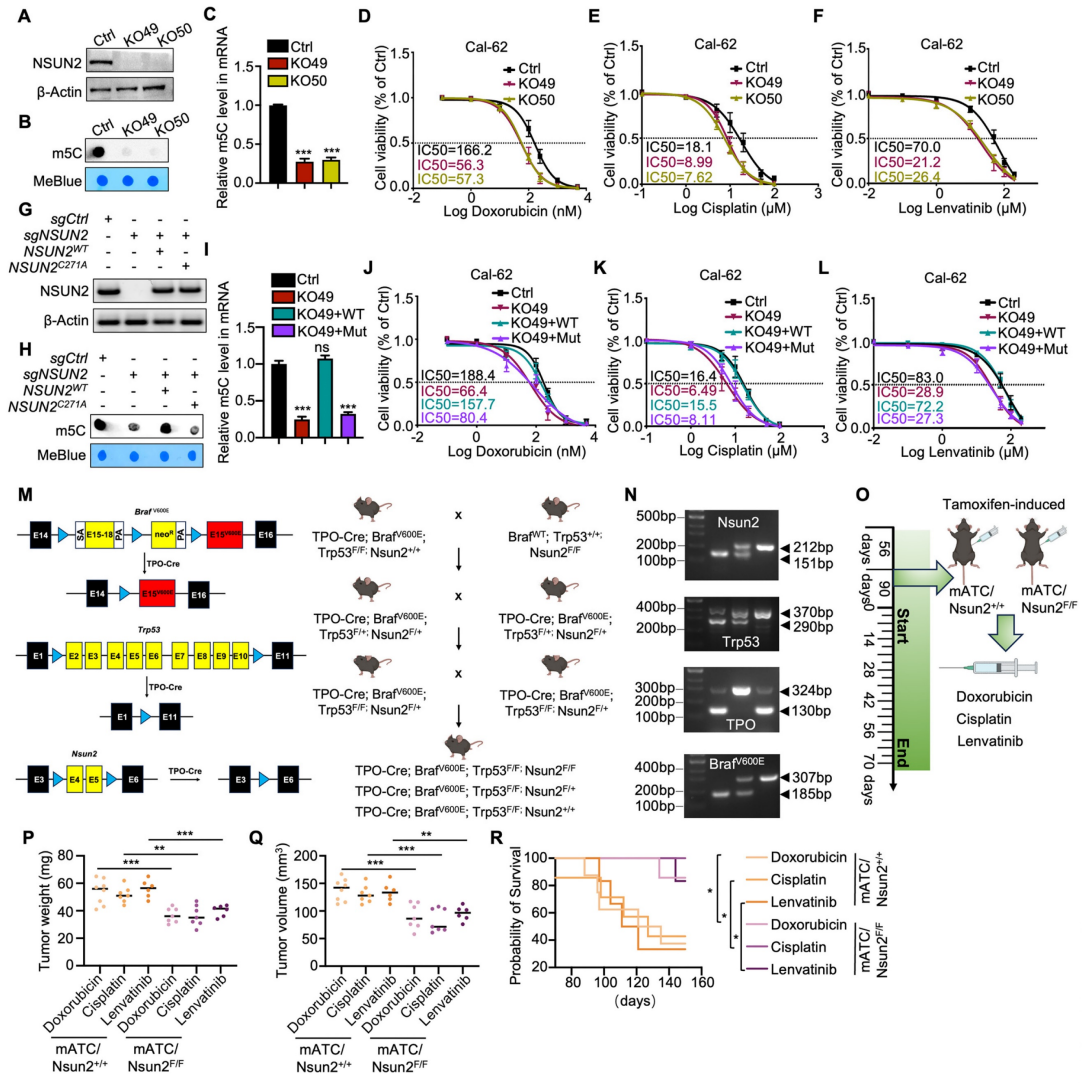
** NSUN2-induced MDR in ATC depends on its methyltransferase activity.** A) Western blot analysis of NSUN2 expression in Cal-62 cells with NSUN2 knockout. B) Dot blot assay examining the effect of NSUN2 knockout on m^5^C levels in mRNA transcriptomes of Cal-62 cells. C) Colorimetric m^5^C quantification assay confirming alterations in m^5^C levels following NSUN2 knockout in Cal-62 cells. D-F) Cell growth inhibition assays evaluating the impact of NSUN2 knockout on Cal-62 cell sensitivity to doxorubicin, cisplatin, and lenvatinib. G) Relative protein expression of NSUN2 in Cal-62 cells after treatment with the indicated vectors. H) Dot blot assay measuring m^5^C abundance in mRNA transcriptomes of Cal-62 cells following vector treatment. I) Colorimetric m^5^C quantification assay confirming m^5^C changes in Cal-62 cells treated with the indicated vectors. J-L) Cell growth inhibition assay assessing IC^50^ values in Cal-62 cells transfected with the indicated vectors. M) Schematic depicting the generation of spontaneous ATC mouse models, illustrating both the targeting strategy (left) and breeding scheme (right). N) Mouse genotyping results, with DNA gel band sizes of 130/324 bp for TPO-cre, 185/307 bp for Braf^V600E^, 290/370 bp for Trp53^flox/flox^, and 151/212 bp for Nsun2^flox/flox^. O) Diagram showing the administration of doxorubicin, cisplatin, and lenvatinib in genetically engineered mice. mATC (murine anaplastic thyroid carcinoma). P) Scatter plot displaying the final tumor weights for the indicated groups. Q) Scatter plot presenting the final tumor volumes for the indicated groups. R) Survival curves for mice with ATC for the indicated groups. The data are presented as the mean ± SD. All *p < 0.05, **p < 0.01, ***p < 0.001.

According to the latest American Thyroid Association Guidelines, chemotherapy drugs (doxorubicin, cisplatin) or tyrosine kinase inhibitors (TKIs) such as lenvatinib are recommended for systemic treatment of ATC. To evaluate the function of NSUN2 in multidrug resistance of ATC, we knocked down NSUN2 in two ATC cell lines, Cal-62 and ACT-1. The knockdown efficiency was determined by western blot analysis (Figure S2A). Dot blot assays revealed a significant decrease in total mRNA m^5^C levels following NSUN2 knockdown (Figure S2B). IC_50_ assays further demonstrated that reduced NSUN2 expression markedly increased the sensitivity of ATC cells to doxorubicin, cisplatin, and lenvatinib (Figure S2C-H). To further validate our findings, NSUN2 was knocked out in Cal-62 cells, and western blot analysis was used to confirm the knockout (Figure 2A). Dot blot analyses and colorimetric m^5^C quantification assays revealed that NSUN2 knockout notably reduced m^5^C levels in total mRNA (Figure 2B-C). Consistent with the knockdown results, the IC_50_ assay showed that NSUN2 knockout cells exhibited significantly increased sensitivity to doxorubicin, cisplatin, and lenvatinib (Figure 2D-F).

NSUN2 function in tumors is largely dependent on its methyltransferase activity. To determine whether NSUN2-mediated MDR in ATC relies on this enzymatic function, we generated an enzymatic-dead NSUN2 mutant by introducing a point mutation at the catalytic residue cysteine 271. We then reintroduced either wild-type or mutant NSUN2 into NSUN2-knockout cells (Figure 2G). Both dot blot analyses and colorimetric m^5^C quantification assays indicated that only wild-type NSUN2 restored m^5^C methyltransferase activity in knockout cells (Figure 2H-I). Moreover, IC_50_ assays demonstrated that wild-type NSUN2, but not the mutant, diminished ATC cell sensitivity to doxorubicin, cisplatin, and lenvatinib (Figure 2J-L).

To further explore the physiological role of NSUN2 in MDR of ATC, we developed a spontaneous tumorigenic model of murine anaplastic thyroid carcinoma (mATC) with an Nsun2 knockout by crossing Nsun2^flox/flox^ mice with TPO-cre;Braf^V600E^;Trp53^ flox/flox^ mice (Figure 2M). Genotyping was confirmed by PCR analysis (Figure 2N). Hematoxylin and eosin (HE) staining confirmed successful tumor formation, and immunohistochemical (IHC) analysis verified NSUN2 depletion (Figure S2I-J). We treated the genetically engineered mice with doxorubicin, cisplatin, or lenvatinib (Figure 2O). Remarkably, compared with controls, the NSUN2 knockout mice developed significantly smaller tumors (Figure 2P-Q) and exhibited longer overall survival (Figure 2R). In summary, these findings demonstrate that NSUN2-driven MDR in ATC depends on its methyltransferase activity.

### NSUN2-mediated m^5^C modification drives alternative splicing reprogramming by targeting SRSF6 in ATC

**Figure 3:**
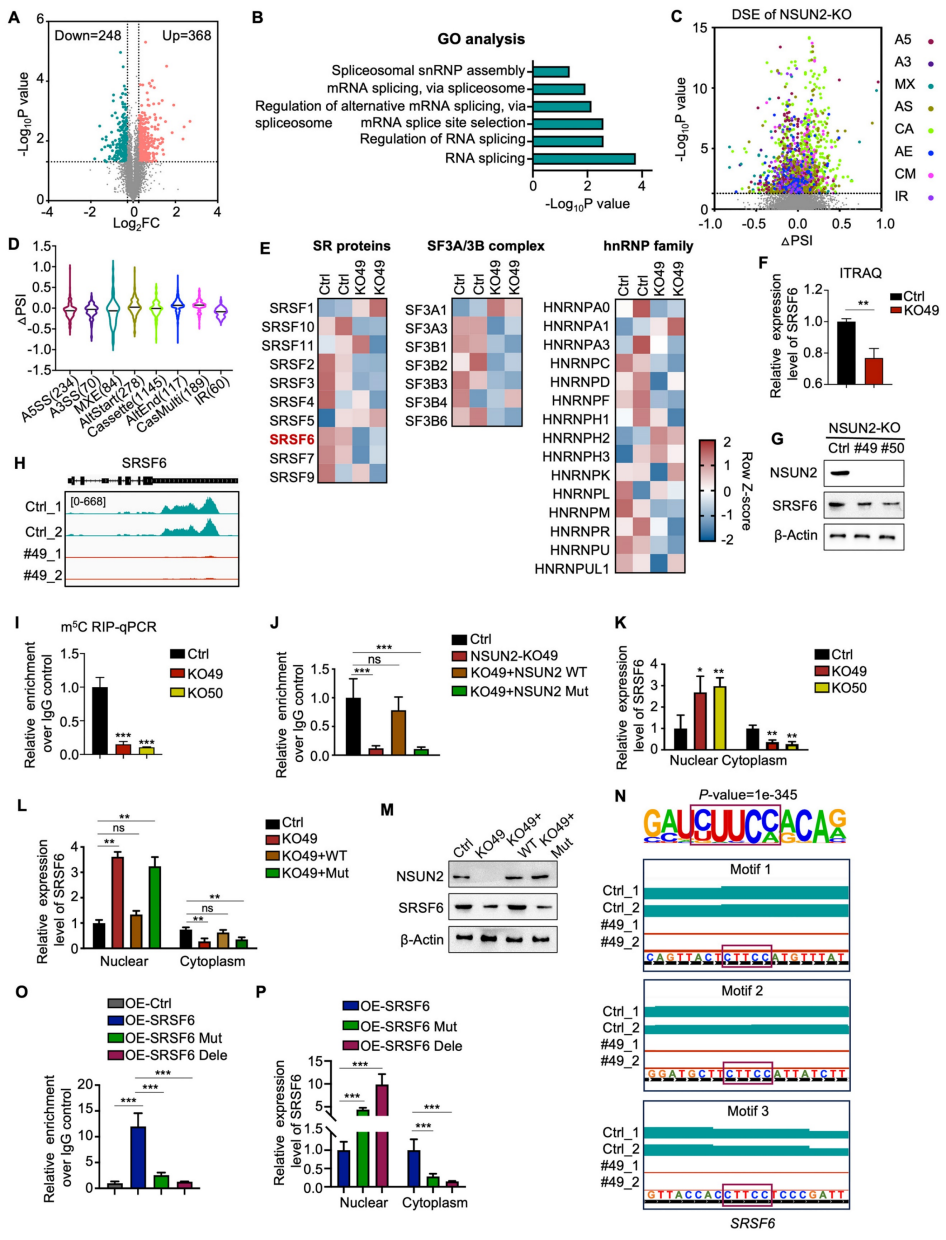
** NSUN2-mediated m^5^C modification drives alternative splicing reprogramming by targeting SRSF6 in ATC.** A) Volcano plot illustrating changes in protein levels in Cal-62 cells following NSUN2 knockout, as determined by TMT-MS. B) GO enrichment analysis of TMT-MS data from Cal-62 cells after NSUN2 knockout. C) Volcano plots showing the percent splicing inclusion (ΔPSI) of differentially spliced genes in Cal-62 cells after NSUN2 knockout. D) Violin plots highlighting significant ΔPSI changes in Cal-62 cells after NSUN2 knockout. E) Heatmap of TMT-MS data indicating differentially expressed genes among SF3A/B, the U2AF core complex, and the hnRNP family in Cal-62 cells following NSUN2 knockout. F) Statistical analysis of SRSF6 protein levels from TMT-MS data in Cal-62 cells following NSUN2 knockout. G) Western blot analysis of the indicated proteins in Cal-62 cells with NSUN2 knockout. H) Integrative Genomics Viewer (IGV) tracks of MeRIP-seq data surrounding the SRSF6 locus in Cal-62 cells after NSUN2 knockout. I) MeRIP-qPCR measuring m^5^C enrichment in SRSF6 mRNA in Cal-62 cells with NSUN2 knockout. J) MeRIP-qPCR assessing m^5^C enrichment in SRSF6 mRNA in Cal-62 cells treated with indicated vectors. K) Nuclear and cytoplasmic distribution of SRSF6 mRNA in Cal-62 cells upon NSUN2 knockout, evaluated by qRT-PCR. L) Nuclear and cytoplasmic distribution of SRSF6 mRNA in Cal-62 cells treated with the indicated vectors. M) Western blot analysis of the indicated proteins in Cal-62 cells treated with the indicated vectors. N) Canonical m^5^C binding sites within SRSF6. Top, motif sequence at m^5^C binding sites; bottom, identified m^5^C sites in SRSF6. O) MeRIP-qPCR quantifying m^5^C enrichment in SRSF6 mRNA in Cal-62 cells treated with the indicated vectors. P) Nuclear and cytoplasmic distribution of SRSF6 mRNA in Cal-62 cells treated with the indicated vectors. The data are presented as the mean ± SD. All *p < 0.05, **p < 0.01, ***p < 0.001.

To elucidate the function and mechanism of NSUN2 in ATC cells, we conducted TMT labeling followed by mass spectrometry (MS) analysis. TMT-MS data identified 368 upregulated and 248 downregulated proteins upon NSUN2 knockout (Figure 3A). Further Gene Ontology (GO) enrichment analyses underscored RNA splicing-related processes, such as “RNA splicing,” “regulation of RNA splicing,” and “mRNA splicing,” as the most prominently affected pathways following NSUN2 knockout (Figure 3B). To achieve robust insights into alternative splicing (AS), we performed RNA-seq and AS analyses on two independently generated NSUN2 knockout cell lines (49 and 50). Analyses revealed 69,150 AS events (Figure 3C), of which 2,177 were significantly regulated by NSUN2. Intriguingly, all AS event types were influenced by NSUN2 knockout, indicating a broad impact on splicing (Figure S3A). Notably, more than half (52.6%) of the differentially spliced events (DSE) were cassette exons (1145), which can profoundly affect the structure and function of resulting proteins (Figure 3D). In addition, GSEA demonstrated that NSUN2 knockout was significantly linked to “multiple drug resistance” and “chemotherapy and TKIs resistance” (Figure S3B-E).

To further investigate the mechanism by which NSUN2 regulates AS in ATC, we evaluated spliceosome-associated proteins (SF3A/B, U2AF core complexes, and hnRNP family) following NSUN2 knockout (Figure 3E). Among these, SRSF6, an oncogenic splicing factor, was the most significantly downregulated in NSUN2 knockout cells (Figure 3F and S3F), which were confirmed by western blot (Figure 3G and S3G-I). However, transcriptome data indicated that the mRNA level of SRSF6 remained unchanged following NSUN2 knockout, a finding confirmed by qRT-PCR (Figure S3J-K). Considering NSUN2 participates in mRNA m^5^C modification, thereby regulating gene expression post-transcriptionally, we hypothesized that SRSF6 mRNA could be a direct substrate of NSUN2. To explore this hypothesis, we conducted MeRIP-seq and discovered a significant downregulation of m^5^C levels in SRSF6 mRNA following NSUN2 knockout (Figure 3H). MeRIP-qPCR further confirmed the observed decrease (Figure 3I). Reintroducing wild-type NSUN2, but not its enzymatic mutant, restored m^5^C levels in SRSF6 mRNA (Figure 3J).

Evidence highlighting m^5^C modification's role in mRNA export regulation, coupled with the reduction in SRSF6 protein levels observed upon NSUN2 knockout without impacting its mRNA, supports the hypothesis that NSUN2 regulates nuclear-cytoplasmic shuttling of SRSF6. Indeed, nuclear-cytoplasmic fractionation followed by qPCR showed an increase in nuclear SRSF6 mRNA and a concurrent reduction in the cytoplasm following NSUN2 knockout, with unchanged total mRNA levels (Figure 3K). Reconstitution with wild-type NSUN2 normalized SRSF6 mRNA export, whereas the mutant had no effect (Figure 3L). The nuclear-cytoplasmic separation was evaluated by qPCR (Figure S3L-M). Correspondingly, only wild-type NSUN2 restored SRSF6 protein (Figure 3M). Motif analysis (HOMER) revealed an enrichment of the CUUCC motif, indicative of m^5^C modification sites, in SRSF6 mRNA. We mutated CUUCC to CUUGG or deleted the motifs (Figure 3N), which substantially decreased m^5^C levels in SRSF6 (Figure 3O) and caused the mutated/deleted forms to accumulate in the nucleus (Figure 3P). The nuclear-cytoplasmic separation was evaluated by qPCR (Figure S3N). In summary, our results indicate that NSUN2-mediated m^5^C modification of SRSF6 promotes its nuclear-to-cytoplasmic shuttling, driving widespread alternative splicing reprogramming in ATC.

### NSUN2 promotes MDR in ATC by enhancing the N-glycosylation of ABC transporters

**Figure 4:**
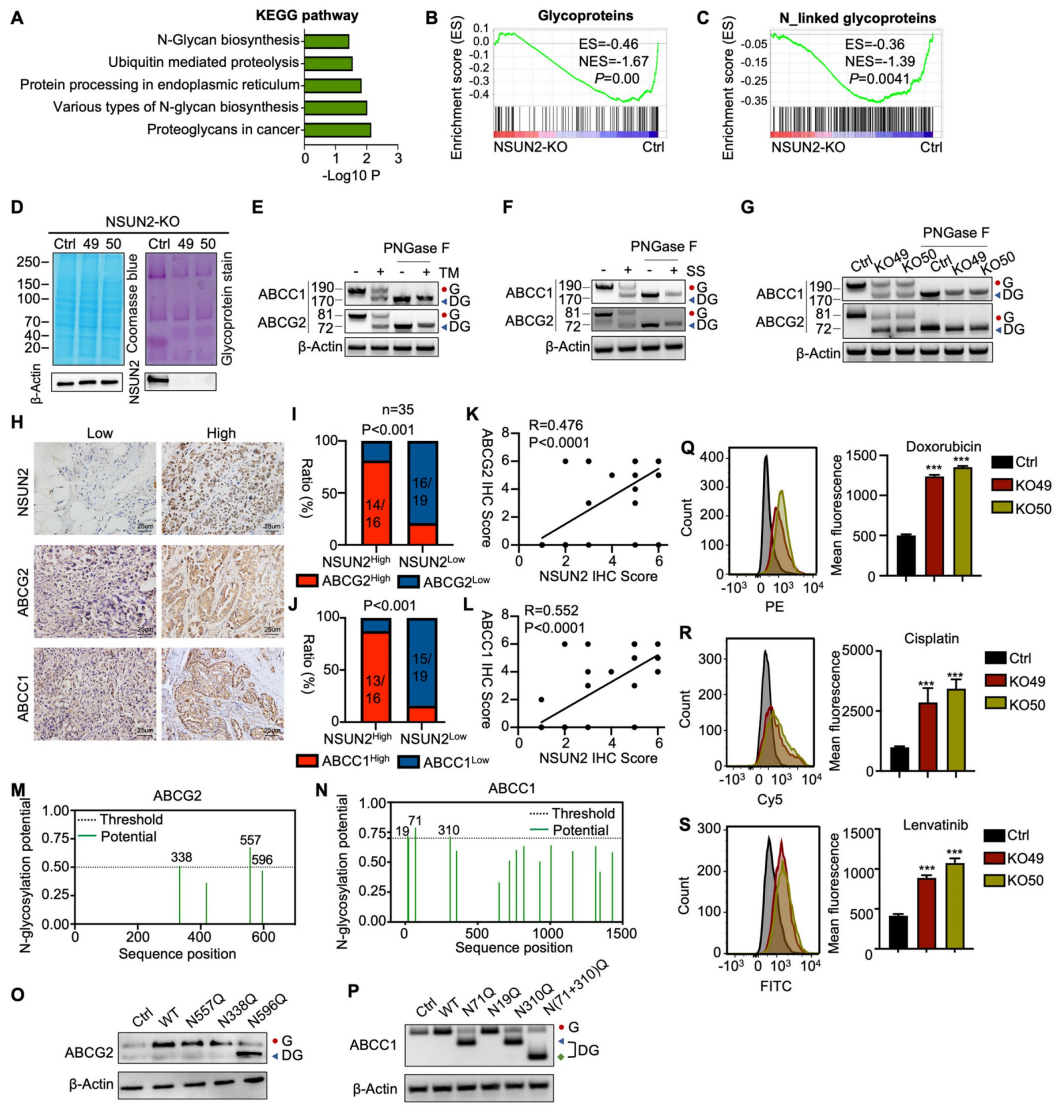
** NSUN2 promotes MDR in ATC by enhancing the N-glycosylation of ABC transporters.** A) Gene Ontology (GO) enrichment analysis of transcriptional data from Cal-62 cells following NSUN2 knockout. B-C) GSEA enrichment analysis of transcriptional data from Cal-62 cells after NSUN2 knockout. D) Glycoprotein staining in Cal-62 cells after NSUN2 knockout is shown on the right. Coomassie blue staining for total protein detection is shown on the left. E-F) Western blotting analysis of indicated protein levels in Cal-62 cells treated with or without tunicamycin (TM), swainsonine (SS). Cell lysate samples were treated with or without PNGase F. “G” means N-glycosylation and “DG” means decreased N-glycosylation. G) Western blot analysis of indicated protein levels in Cal-62 cells with NSUN2 knockout. Cell lysates were treated with or without PNGase F. H) Representative IHC images of NSUN2, ABCG2, and ABCC1 expression in ATC tissues (n = 35). I-J) The chi-square test was used to analyze the correlation between NSUN2 and ABCG2 or ABCC1 expression. K-L) Correlation analysis of IHC scores for NSUN2 and ABCG2 or ABCC1. M-N) The potential N-Glycosylated asparagine sites of ABCG2 and ABCC1 predicted with NetNglyc server. O-P) ABCG2 and ABCC1 expression were detected by western blot in Cal-62 cells transfected with the N557Q, N388Q, N596Q, or N71Q, N19Q, N310Q, N(71+310)Q mutant plasmids, respectively. Q-S) Flow cytometry analysis of intracellular accumulation of doxorubicin, cisplatin, and lenvatinib in Cal-62 cells following NSUN2 knockout. Quantification of mean fluorescence intensity is shown on the right. The data are presented as the mean ± SD. All *p < 0.05, **p < 0.01, ***p < 0.001.

KEGG pathway analysis of RNA-seq data revealed that NSUN2 is involved in N-glycan biosynthesis (Figure 4A), a finding corroborated by GSEA, which showed significant enrichment of glycoprotein and N-linked glycoprotein-related processes after NSUN2 knockout (Figure 4B-C). Glycoprotein staining further demonstrated markedly reduced glycan levels in NSUN2-knockout cells (Figure 4D). To distinguish N-glycosylation from O-glycosylation, we treated ATC cells with the O-glycosylation inhibitor Benzyl-α-N-acetylgalactosamine (BAG), but observed no substantial changes in the key ABC transporter components ABCC1 and ABCG2 (Figure S4A).

Previous studies have shown that N-glycosylation is pivotal for the expression of ABC transporters 23. We thus treated ATC cells with tunicamycin (TM), a GlcNAc phosphotransferase inhibitor that blocks the formation of N-glycosidic bonds, and swainsonine (SS), which completely inhibits mammalian Golgi α-mannosidase II.

Because an N-linked glycosylation site typically adds approximately 3-4 kDa, western blot analysis revealed a slower-migrating band for ABCC1 and ABCG2 in TM- or SS-treated Cal-62 cells, confirming that ABCC1 and ABCG2 are modified by N-linked glycosylation (Figure 4E-F). To analyze, quantitatively, their effects on the protein-expression levels of ABCC1 and ABCG2, N-glycosidase F (PNGase F) was used to remove N-linked glycans. Consistently, western blot analysis showed that ABCC1 and ABCG2 exhibited reduced molecular weight and overall protein levels upon NSUN2 knockout, suggesting a regulatory role for NSUN2 in their N-linked glycosylation (Figure 4G). In line with this, immunohistochemistry of ATC tissues confirmed a strong positive correlation between NSUN2 expression and that of ABCC1 and ABCG2 (Figure 4H-L).

N-glycosylation typically occurs on asparagine residues within the Asn-Xaa-Ser/Thr sequence, with Xaa representing any amino acid except proline. Using the NetNglyc server, we identified potential N-glycosylation sites on ABCG2 and ABCC1 (Figure 4M-N). Site-directed mutagenesis (N→Q) pinpointed N596 of ABCG2 and N71, N310 of ABCC1 as critical for N-glycosylation (Figure 4O-P). Furthermore, simultaneous mutation of N71 and N310 in ABCC1 [N(71+310)Q] led to an even smaller molecular weight shift compared to single mutations, confirming both residues as key N-glycosylation sites (Figure 4P). As ABC transporters contribute to MDR in tumors by mediating drug efflux 32. Therefore, in our experiments, we incubated cells with drug-containing medium for 6 hours, replaced it with drug-free medium, and continued incubation for 6 more hours. Flow cytometry revealed that NSUN2 knockout markedly reduced drug efflux, as evidenced by higher intracellular retention of doxorubicin, cisplatin, and lenvatinib (Figure 4Q-S). In summary, our findings revealed that NSUN2 promotes MDR in ATC by enhancing the N-glycosylation of ABC transporters.

### NSUN2 enhances ABC transporters stability through N-glycosylation

**Figure 5:**
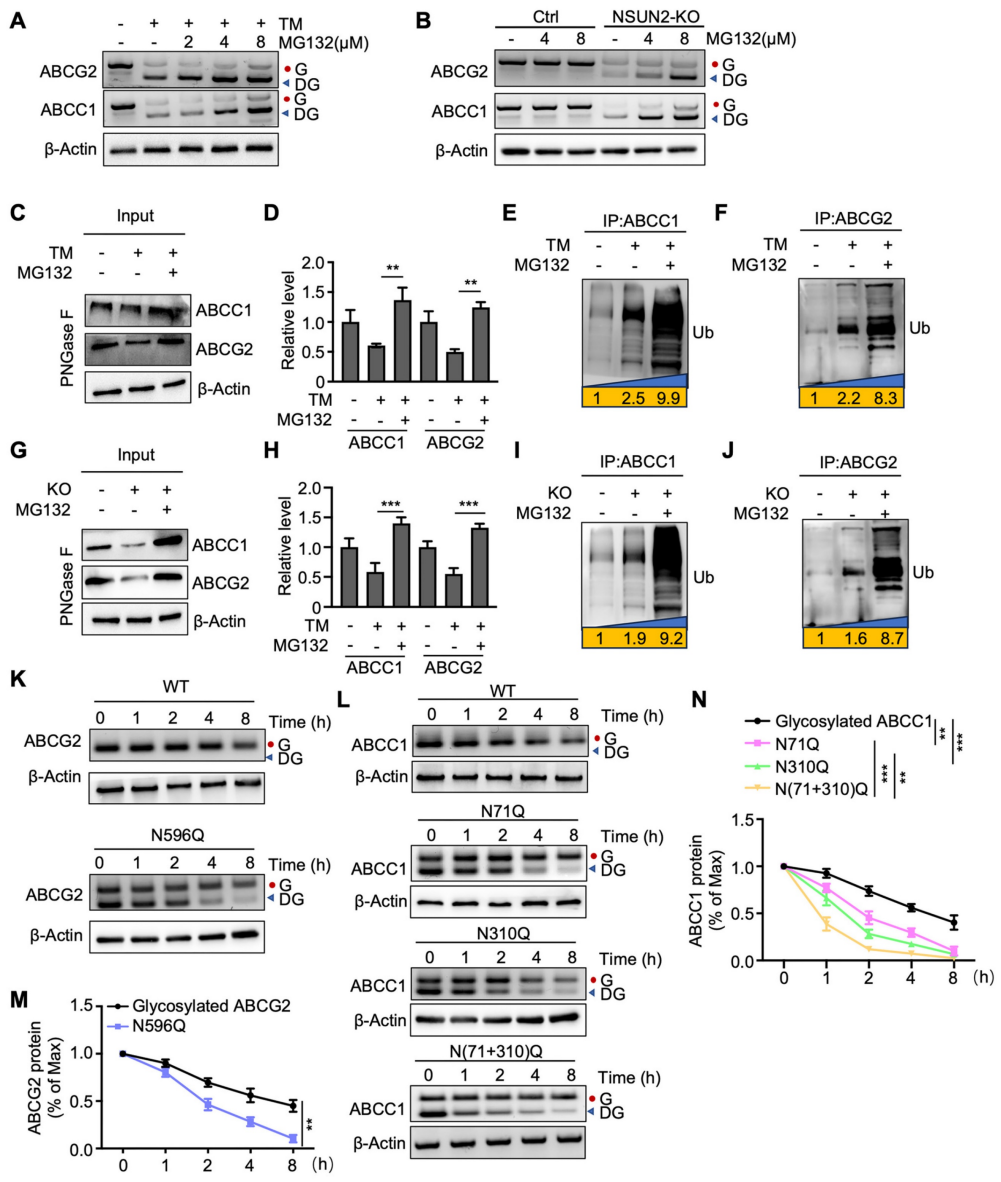
** NSUN2 enhances ABC transporters stability through N-glycosylation.** A) Cal-62 cells were treated with TM or the indicated concentrations of MG132, followed by western blot analysis using the specified antibodies. B) Cal-62 cells with NSUN2 knockout were treated with indicated concentrations of MG132, followed by western blot analysis with indicated antibodies. C) Western blotting analysis of indicated protein levels in Cal-62 cells treated with TM and MG132. Cell lysate samples were treated with PNGase F. D) Quantification of ABCC1 and ABCG2 protein levels performed using ImageJ; data are presented as mean ± SD. E-F) Cal-62 cells treated with TM and MG132 were subjected to ABCC1 or ABCG2 immunoprecipitation (IP), followed by western blot analysis with anti-ubiquitin antibodies. G) Western blotting analysis of indicated protein levels in Cal-62 cells having NSUN2 knockout and treated with MG132. Cell lysate samples were treated with PNGase F. H) Quantification of ABCC1 and ABCG2 protein intensities using ImageJ; data are presented as mean ± SD. I-J) Cal-62 cells having NSUN2 knockout and treated with MG132 were subjected to ABCC1 or ABCG2 immunoprecipitation (IP), followed by western blot analysis with anti-ubiquitin antibodies. K-L) Wild-type (WT) or the indicated ABCG2/ABCC1 mutants expressed in Cal-62 cells were treated with 125 μg/mL cycloheximide (CHX) for specified intervals and analyzed by western blot. M-N) Protein intensities of ABCG2 or ABCC1 were quantified using Image J and normalized to β-Actin levels. The data are presented as the mean ± SD. All *p < 0.05, **p < 0.01, ***p < 0.001.

We then investigated whether deglycosylated ABC transporters are more susceptible to ubiquitin-dependent degradation. The results showed that deglycosylated ABCC1 and ABCG2 were increased upon protease inhibitor MG132 treatment when ABC transporters were deglycosylated by TM (Figure 5A). A similar increase was observed in NSUN2-knockout cells treated with MG132 (Figure 5B). In parallel, MG132 treatment partially rescued the TM-induced downregulation of both ABC transporters (Figure 5C-D). Co-immunoprecipitation of ABCC1 and ABCG2, followed by western blotting with an anti-ubiquitin antibody, further confirmed that TM-induced deglycosylation significantly enhanced ubiquitination of ABCC1 and ABCG2, an effect amplified by MG132 (Figure 5E-F). Consistently, MG132 also mitigated the loss of ABCC1 and ABCG2 in NSUN2-knockout cells (Figure 5G-H), and NSUN2 knockout itself increased ubiquitylated ABCC1 and ABCG2 levels (Figure 5I-J). To test how N-glycosylation site mutations affect ABCG2 and ABCC1 stability, we treated Cal-62 cells with cycloheximide (CHX, 125 µg/mL) and monitored protein turnover. The N596 mutation in ABCG2 and the N71, N310 mutations in ABCC1 shortened protein half-lives relative to fully glycosylated counterparts (Figure 5K-N). Taken together, these findings indicate that NSUN2 enhances ABC transporter stability via N-glycosylation, thus limiting their ubiquitin-dependent degradation.

### NSUN2 promotes N-glycosylation by mediating the alternative splicing of UAP1 through SRSF6

**Figure 6:**
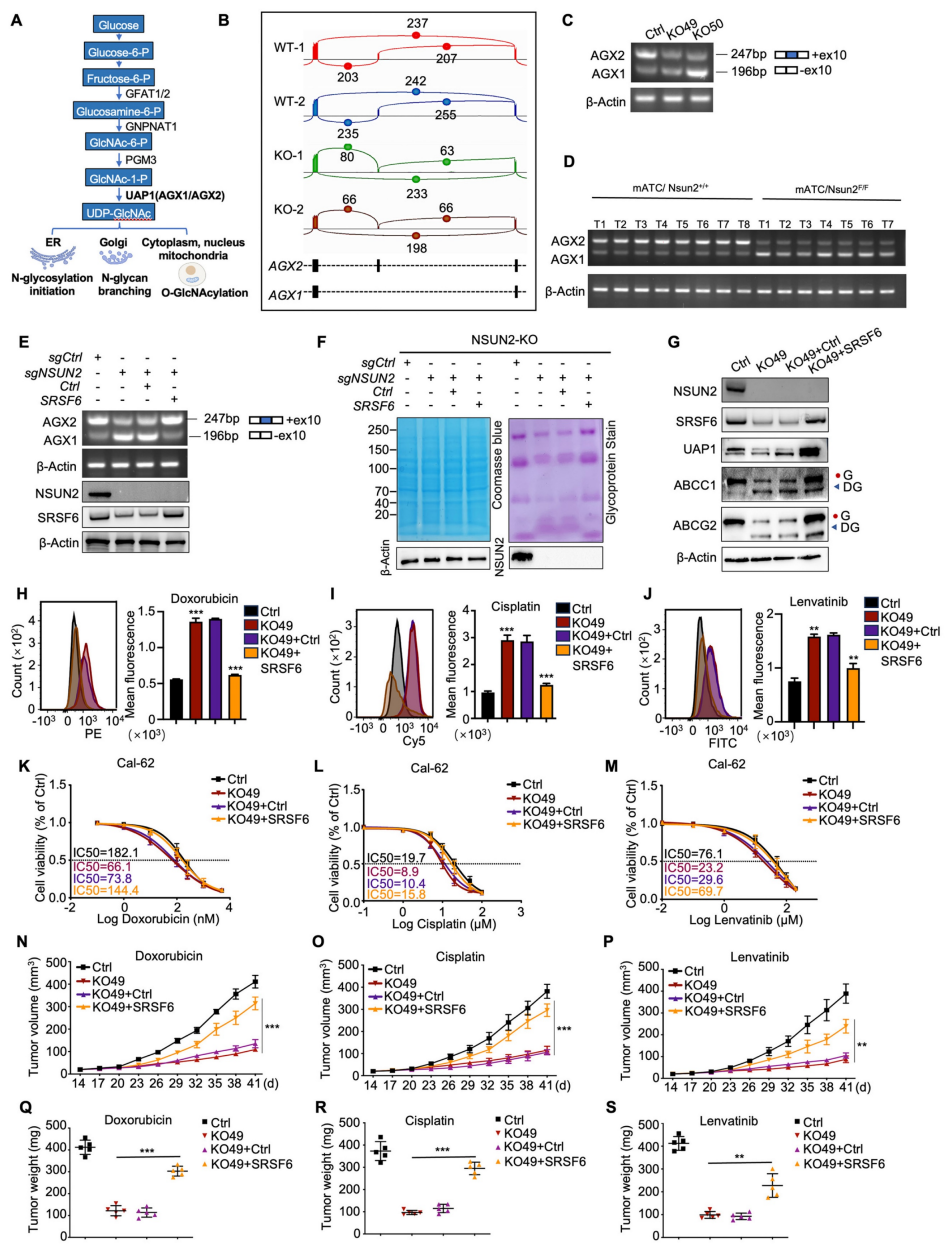
** NSUN2 promotes N-glycosylation by mediating the alternative splicing of UAP1 through SRSF6.** A) UAP1 promotes the biosynthesis of UDP-GlcNAc, a key donor in glycosylation reactions, particularly in N-glycosylation and O-GlcNAc modifications. B) Sashimi plots illustrating changes in UAP1 splicing events in Cal-62 cells following NSUN2 knockout. C) RT-PCR analysis of UAP1 splicing in control versus NSUN2 knockout Cal-62 cells. The number of skipped exons is depicted for each transcript. D) RT-PCR showing the splicing events of UAP1 in spontaneous mouse ATC tumors with or without Nsun2 knockout. E) RT-PCR showing the splicing events of UAP1 in Cal-62 cells treated with indicated vectors (upper panel). The number of skipped exons is depicted for each transcript. The reconstruction of SRSF6 was determined by western blot (lower panel). F) Glycoprotein staining in Cal-62 cells treated with indicated vectors is shown on the right. Coomassie blue staining for total protein detection is shown on the left. G) Western blot analysis of indicated protein levels in Cal-62 cells treated with indicated vectors. H-J) Flow cytometry analysis of intracellular accumulation of doxorubicin, cisplatin, and lenvatinib in Cal-62 cells treated with indicated vectors. Quantification of mean fluorescence intensity is shown on the right. K-M) Cell growth inhibition assay evaluating the effects of the indicated vectors on Cal-62 cell sensitivity to doxorubicin, cisplatin, and lenvatinib. N-P) Tumor growth curves evaluating the impact of the indicated vectors treatment on Cal-62 cells response to doxorubicin, cisplatin, and lenvatinib. Q-S) Tumor final weights evaluating the impact of the indicated vectors treatment on Cal-62 cells response to doxorubicin, cisplatin, and lenvatinib. The data are presented as the mean ± SD. All *p < 0.05, **p < 0.01, ***p < 0.001.

UAP1 is a crucial enzyme in UDP-GlcNAc biosynthesis, a key donor in glycosylation reactions, especially N-glycosylation and O-GlcNAc modifications (Figure 6A). Human UAP1 has two isoforms, AGX1 and AGX2, arising from alternative splicing of a single gene. AGX2 is characterized by an additional 17 amino acid peptide and exhibits higher activity with GlcNAc-1-P, the substrate for UDP-GlcNAc conversion 33. Using the CASH software to analyze differential splicing events, we found that NSUN2 knockout reduced the proportion of UAP1 transcripts containing a specific 51-bp exon, leading to a shift from the AGX2 to the AGX1 isoform (Figure 6B). This result was confirmed by RT-PCR (Figure 6C). Consistently, in spontaneous mouse ATC tumors, UAP1 splicing shifted predominantly to AGX1 in Nsun2-knockout tumors (Figure 6D). To determine whether NSUN2 regulates UAP1 splicing through SRSF6, we overexpressed SRSF6 in NSUN2-knockout cells. SRSF6 overexpression reversed the exon-skipping event, restoring splicing toward the AGX2 isoform (Figure 6E). Furthermore, inhibiting SRSF6 activity via zinc ions (Zn²⁺) or indacaterol further promoted the splicing toward the AGX1 isoform (Figure S5A-B), mirroring the effects observed upon SRSF6 knockdown (Figure S5C-D). A previous study identified a TGGAG motif that serves as a binding site for SRSF6 34. Examination of the UAP1 sequence revealed TGGAC repeats within its tenth exon (Figure S5E), and RIP-qPCR assays confirmed that SRSF6 binds to UAP1 (Figure S5F).

Glycoprotein staining showed that SRSF6 overexpression also rescued N-glycosylation levels (Figure 6F), and western blot analysis confirmed the recovery of N-glycosylation in ABCG2 and ABCC1 (Figure 6G). Furthermore, in ATC tissues, SRSF6 levels correlated positively with the expression of these ABC transporters, suggesting that NSUN2-driven multidrug resistance may be mediated via SRSF6 (Figure S5G-J). Flow cytometry demonstrated that overexpressing SRSF6 in NSUN2-knockout cells restored drug efflux and decreased the intracellular accumulation of doxorubicin, cisplatin, and lenvatinib (Figure 6H-J). Accordingly, IC_50_ assays demonstrated that SRSF6 overexpression significantly diminished the sensitivity of Cal-62 cells to these drugs (Figure 6K-M), whereas SF3B3 overexpression had no comparable effect (Figure S5K-N). Moreover, *in vivo* experiments demonstrated that SRSF6 overexpression reversed the enhanced drug sensitivity observed in NSUN2-knockout cells, leading to tumor growth and final tumor weights comparable to those of NSUN2-knockout controls (Figure 6N-S). Collectively, these results establish that NSUN2 promotes N-glycosylation by modulating the alternative splicing of UAP1 via SRSF6.

### ALYREF functions as an m^5^C “reader” that recognizes and shuttles SRSF6 from nucleus to cytoplasm

**Figure 7:**
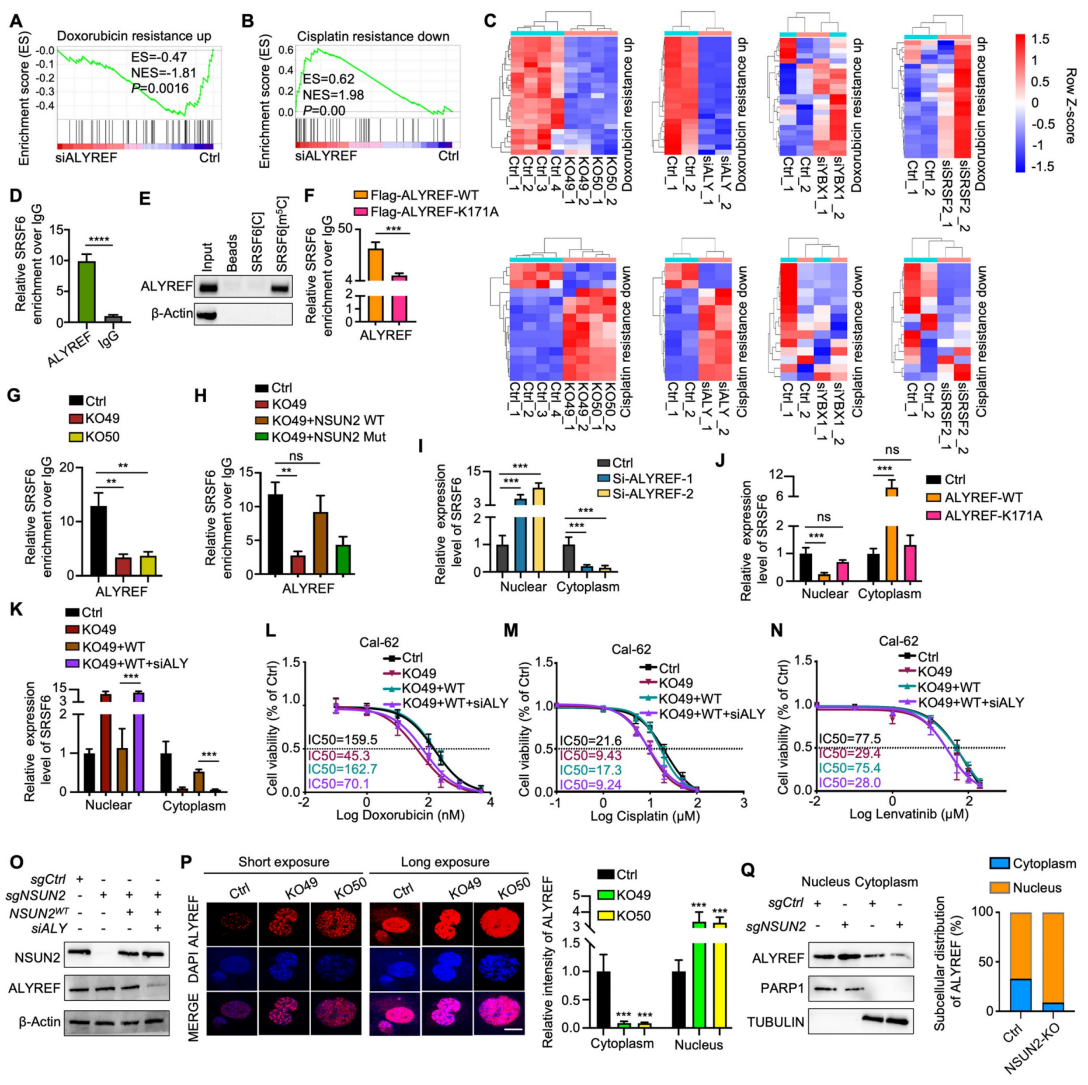
** ALYREF functions as an m^5^C “reader” that recognizes and shuttles SRSF6 from nucleus to cytoplasm.** A-B) GSEA enrichment analysis indicating that ALYREF expression correlates with chemotherapy resistance. C) Heatmap depicting genes associated with doxorubicin and cisplatin resistance in Cal-62 cells with NSUN2 knockout or ALYREF, YBX1, and SRSF2 knockdown. D) RIP-qPCR assay detecting ALYREF binding to SRSF6 in Cal-62 cells. E) Western blotting analysis of the indicated proteins obtained from SRSF6[m^5^C] pulldown assay. F) RIP-qPCR comparing ALYREF-WT and ALYREF-K171A binding to SRSF6 in Cal-62 cells treated with indicated vectors. G) RIP-qPCR assessing ALYREF binding to SRSF6 mRNA in Cal-62 cells following NSUN2 knockout. H) RIP-qPCR evaluating ALYREF binding to SRSF6 mRNA in Cal-62 cells treated with indicated vectors. I) Nuclear and cytoplasmic distribution of SRSF6 mRNA in Cal-62 cells with ALYREF knockdown, evaluated by qRT-PCR assay. J) Nuclear and cytoplasmic distribution of SRSF6 mRNA in Cal-62 cells overexpressing ALYREF or ALYREF-K171A mutant. K) Nuclear and cytoplasmic distribution of SRSF6 mRNA in Cal-62 cells treated with indicated vectors. L-N) Cell growth inhibition assay evaluating the impact of indicated vectors treatment on Cal-62 cell response to doxorubicin, cisplatin, and lenvatinib. O) Western blotting analysis of the indicated protein levels in Cal-62 cells treated with indicated vectors. P) Immunofluorescence staining of ALYREF in NSUN2-knockout Cal-62 cells (left) and quantitative analysis of nuclear/cytoplasmic ALYREF distribution (right). Q) Western blotting analysis (left) and corresponding quantification (right) of nuclear and cytoplasmic ALYREF in control and NSUN2-knockout Cal-62 cells are shown, with PARP1 and TUBULIN as nuclear and cytoplasmic markers, respectively. The data are presented as the mean ± SD. All *p < 0.05, **p < 0.01, ***p < 0.001.

Notably, m^5^C-methylated RNAs are often recognized by specific reader proteins 5. To identify potential m^5^C methylation readers for SRSF6 mRNA, we knocked down ALYREF, YBX1, and SRSF2 and performed RNA-seq, confirming knockdown efficiency by qRT-PCR (Figure S6A-C). GSEA revealed a significant association between ALYREF knockdown and “doxorubicin resistance” and “cisplatin resistance,” mirroring the effects of NSUN2 knockout (Figure 7A-B). Furthermore, ALYREF knockdown led to similar alterations in genes related to drug resistance, indicating that ALYREF serves as the primary m^5^C reader of NSUN2 mediated MDR in ATC (Figure 7C). RIP-qPCR analysis confirmed that ALYREF directly binds to SRSF6 in ATC cells (Figure 7D), which was further validated by RNA pull-down experiments (Figure 7E). A previous report indicates that the ALYREF K171A mutation significantly reduces its binding affinity for m^5^C-modified oligonucleotides 30. Accordingly, overexpressing the K171A mutant in Cal-62 cells demonstrated that this mutation markedly diminishes ALYREF binding to m^5^C-SRSF6 (Figure 7F). Consistently, ALYREF-RIP-qPCR demonstrated reduced ALYREF binding to SRSF6 in NSUN2-knockout cells, which was rescued by reintroducing wild-type NSUN2 but not its m^5^C-transferase-defective mutant (Figure 7G-H). These observations suggest that ALYREF shuttles m^5^C-modified SRSF6 mRNA from the nucleus to the cytoplasm under the regulation of NSUN2. Indeed, ALYREF knockdown significantly impaired nuclear-to-cytoplasmic transport of SRSF6, while overexpression of wild-type ALYREF (but not the K171 mutant) restored this process (Figure 7I-J). Notably, ALYREF knockdown reversed the NSUN2 overexpression-induced increase in SRSF6 export, as confirmed by nuclear-cytoplasmic fractionation and qPCR (Figure 7K). The nuclear-cytoplasmic separation was evaluated by qPCR (Figure S6D-F). Moreover, an IC_50_ assay demonstrated that ALYREF knockdown mitigates the MDR induced by NSUN2 overexpression (Figure 7L-N), and its efficacy of knockdown was verified by western blot (Figure 7O). Assessments of ALYREF subcellular localization revealed a substantial increase in nuclear staining and decreased cytoplasmic staining upon NSUN2 knockout, despite unchanged total protein levels (Figure 7P and S6G). Accordingly, ALYREF exhibited enhanced nuclear retention and reduced cytoplasmic localization in NSUN2-deficient cells (Figure 7Q). Taken together, these findings identify ALYREF as a classical m^5^C reader that recognizes and mediates the nuclear export of m^5^C-modified SRSF6 mRNA, contributing to MDR in ATC.

### Small-molecule NSUN2 inhibitor enhances multidrug sensitivity in ATC

**Figure 8:**
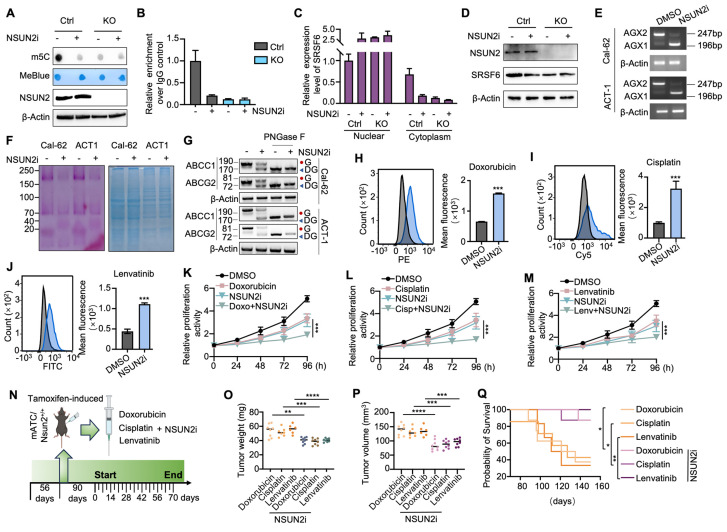
** Small-molecule NSUN2 inhibitor enhances multidrug sensitivity in ATC.** A) Dot blot analysis assessing m^5^C levels in mRNA from Cal-62 cells with NSUN2 knockout, with or without NSUN2 inhibitor treatment. B) RT-qPCR-based MeRIP demonstrating m^5^C enrichment in SRSF6 mRNA in Cal-62 cells with NSUN2 knockout, with or without NSUN2 inhibitor. C) Nuclear and cytoplasmic distribution of SRSF6 mRNA in Cal-62 cells with NSUN2 knockout, with or without NSUN2 inhibitor, evaluated by qRT-PCR assay. D) Western blot analysis of indicated protein levels in Cal-62 cells with NSUN2 knockout, with or without NSUN2 inhibitor. E) RT-PCR shows the splicing events of UAP1 in Cal-62 cells and ACT-1 cells treated with or without NSUN2 inhibitor. F) Glycoprotein staining in Cal-62 cells and ACT-1 cells treated with or without NSUN2 inhibitor (right). Coomassie blue staining for total protein detection is shown on the left. G) Western blot analysis of indicated protein levels in Cal-62 cells and ACT-1 cells treated with or without NSUN2 inhibitor. Cell lysates were treated with or without PNGase F. H-J) Flow cytometry analysis of intracellular accumulation of doxorubicin, cisplatin, and lenvatinib in Cal-62 cells treated with or without NSUN2 inhibitor. Quantification of mean fluorescence intensity is shown on the right. K-M) Cell growth inhibition assay assessing the effect of combining NSUN2 inhibitor with doxorubicin, cisplatin, or lenvatinib on Cal-62 cells. N) Schematic illustrating the administration of NSUN2 inhibitor, doxorubicin, cisplatin, and lenvatinib in genetically engineered mice. O) Scatter plot depicting final tumor weights in the specified groups. P) Scatter plot showing final tumor volumes in the specified groups. Q) Survival curves for mice with ATC across the indicated groups. The data are presented as the mean ± SD. All *p < 0.05, **p < 0.01, ***p < 0.001.

Given NSUN2's role in multidrug resistance (MDR), we hypothesized that targeting NSUN2 could be a promising therapeutic strategy for ATC. Guided by previously reported structural insights, we designed a small-molecule NSUN2 inhibitor (NSUN2i) that interacts with key NSUN2 residues, including Tyr83, Hie86, Asn157, Gln605, and Glu694 35. The 2D structure is shown in Figure S7A. Dot-blot assay demonstrated reduced m^5^C abundance following NSUN2i treatment in ATC cells (Figure 8A). Consistent with NSUN2 knockout results, MeRIP-qPCR confirmed that NSUN2i markedly reduced m^5^C levels in SRSF6, thereby impeding its nuclear export without altering overall mRNA levels (Figure 8B-C and S7B). Western blot analysis further revealed decreased SRSF6 protein expression under NSUN2i treatment (Figure 8D). Additionally, NSUN2i promoted a shift in UAP1 splicing from the AGX2 to the AGX1 isoform, a change that attenuated N-glycosylation in Cal-62 and ACT-1 cells (Figure 8E-F). Western blot confirmed that NSUN2i reduced the levels of N-glycosylated ABC transporters (Figure 8G). Flow cytometry indicated that intracellular accumulation of doxorubicin, cisplatin, and lenvatinib was significantly elevated by NSUN2i (Figure 8H-J and S7C-E), and combination index (CI) analysis showed strong synergy (CI < 1) between NSUN2i and these agents (Figure S7F-G). Likewise, CCK-8 assays demonstrated heightened drug sensitivity in Cal-62 and ACT-1 cells (Figure 8K-M and S7H-J). To investigate *in vivo* synergy, we treated spontaneous tumorigenic ATC mice with NSUN2i combined with doxorubicin, cisplatin, or lenvatinib (Figure 8N). The combination therapy significantly enhanced tumor growth inhibition and prolonged animal survival compared to each agent alone (Figure 8O-Q). Collectively, these data highlight NSUN2 as a promising therapeutic target for combination cancer treatments involving chemotherapy or TKIs.

## Discussion

**Figure 9:**
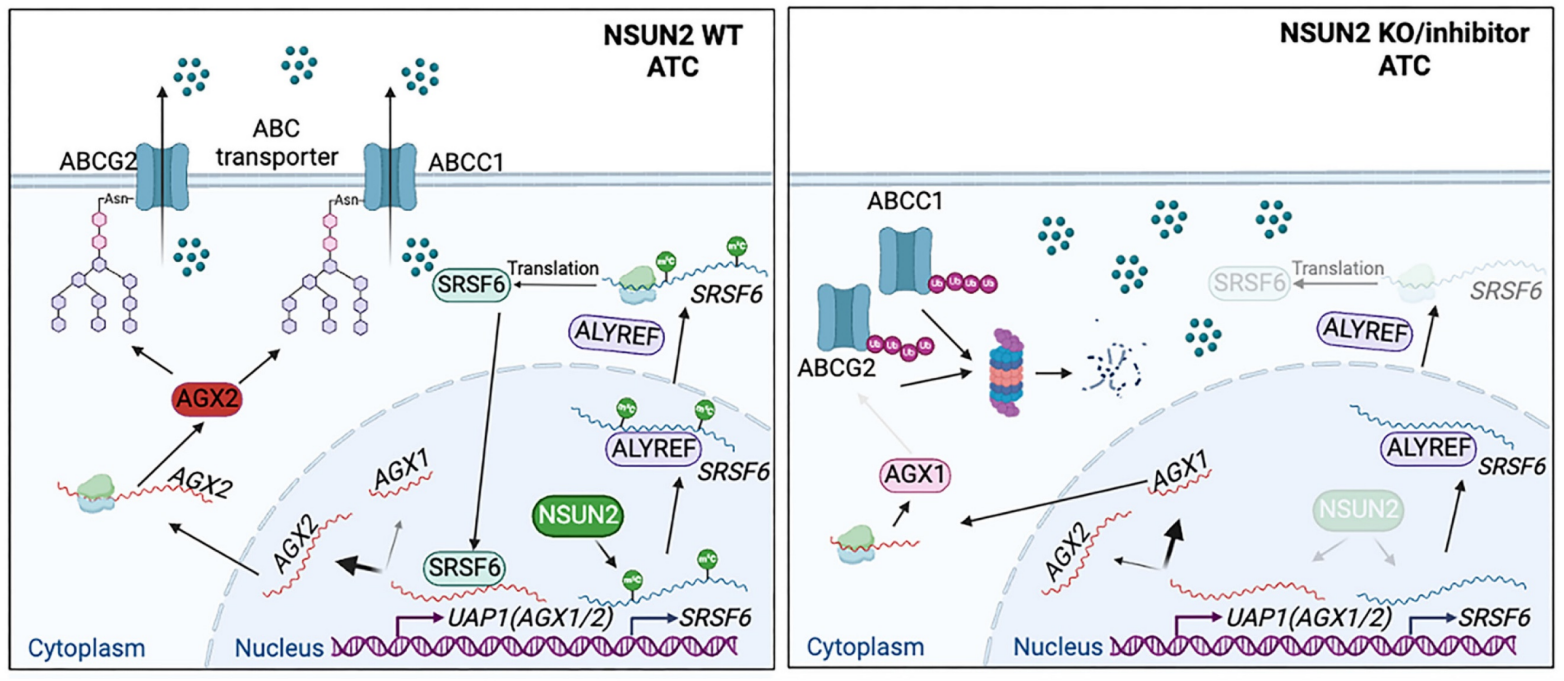
Schematic model of the function and mechanism of NSUN2 in ATC multidrug resistance.

Our study highlights that NSUN2-mediated mRNA m^5^C modification of SRSF6 reprograms alternative splicing in ATC. Specifically, it shifts UAP1 splicing from the AGX1 to AGX2 isoform, thereby enhancing N-linked glycosylation of ABC transporters and preventing their ubiquitin-dependent degradation (Figure 9). Consequently, NSUN2 emerges as a promising target for combatting multidrug resistance (MDR) in ATC.

Accumulating evidence links aberrant m^5^C modification to oncogenesis and progression in various cancers, including colorectal, gynecologic, breast, and pancreatic cancers 36-39. As a well-defined RNA methyltransferase, NSUN2 mediates m^5C modification and promotes tumorigenesis by regulating RNAs such as ENO1, TIAM1, HGH1, NRF2, and LIN28B 40-44. Elevated NSUN2 expression in multiple malignancies is correlated with lymph node metastasis, advanced clinical stage, and reduced overall survival 45. Moreover, in bulk and single-cell RNA sequencing analyses of ATC samples, higher NSUN2 expression shows a strong association with chemoresistance and reduced sensitivity to tyrosine kinase inhibitors. Consistent with these observations, NSUN2 knockout lowers the IC_50 of doxorubicin, cisplatin, and lenvatinib in ATC cells. In genetically engineered mouse models, NSUN2 deletion significantly curtails tumor growth and improves survival upon chemotherapy and tyrosine kinase inhibitor treatment. Taken together, our findings underscore the pivotal role of NSUN2 in driving MDR in ATC.

To elucidate the molecular mechanisms by which NSUN2 promotes MDR in ATC, we performed proteomic analyses and observed a marked enrichment of mRNA splicing-related processes following NSUN2 knockout. Among these, SRSF6 was identified as a critical downstream target, regulated in an m^5^C-dependent manner. SRSF6, a highly conserved RNA-binding splicing factor, has been implicated in tumorigenesis across various cancers via canonical alternative splicing pathways 46, 47. In the present study, we show that NSUN2-mediated m^5^C modification of SRSF6 underlies the reprogramming of alternative splicing in ATC. Generally, m^5^C-modified mRNAs are recognized by reader proteins, enabling functions like regulating mRNA nuclear-cytoplasmic shuttling in an m^5^C-dependent manner 30, 48. Several m^5^C reader proteins have been identified to date, including YBX1, SRSF2, and ALYREF. As m^5^C reader proteins responsible for different functions, YBX1 recognizes m^5^C-modified mRNA to maintain its stability, SRSF2 binds m^5^C-modified mRNA leading to alternative splicing events, and ALYREF facilitates its target mRNA export in an m^5^C-dependent manner 30, 40, 49. To identify the reader of m^5^C-SRSF6, we conducted mass spectrometry analysis and found that ALYREF is responsible for the SRSF6 mRNA export in an m^5^C-dependent manner. Consistent with this, as an m^5^C reader protein, ALYREF has been found in various types of tumors to promote tumor progression by mediating the nucleocytoplasmic transport of substrate mRNAs 50-52. In parallel, we focused on UAP1, an essential enzyme in UDP-GlcNAc biosynthesis that supplies a crucial substrate for glycosylation reactions, especially N-glycosylation 53, 54. UAP1 exists in two isoforms, AGX1 and AGX2, via alternative splicing. AGX2 carries an additional 17 amino acids, rendering it more active toward GlcNAc-1-P 55. Our findings show that SRSF6 directs the splicing of UAP1 from AGX1 to AGX2, thereby enhancing N-glycosylation in ATC. Collectively, these data establish NSUN2 and ALYREF as the “writer” and “reader” of SRSF6 m^5^C modification, respectively, driving the alternative splicing of UAP1 toward the AGX2 isoform and increasing N-glycosylation in ATC.

Substantial evidence suggests that the expression of ABC transporters can confer tumor resistance to chemotherapy and targeted therapy by actively transporting substrates across cell membranes 56-58. Previous research has highlighted ABC transporters as potential mechanisms behind multidrug resistance in ATC. Specifically, studies evaluating 10 out of 49 known ABC transporters in ATC have revealed that ABCC1 and ABCG2 exhibit the highest expression levels in ATC tissues 2. Meanwhile, owning to N-linked glycosylation is known to enhance the stability of glycosylated proteins compared to their non-glycosylated counterparts 59-61. Research shows that disrupting N-linked glycosylation enhances ubiquitin-mediated proteasomal degradation of ABCG2 22. Additionally, study found that increased levels of glycosylation-defective ABCC1 are associated with resistance to platinum compounds in ovarian carcinoma cells 62. Therefore, we selected ABCC1 and ABCG2 as the main focus of our investigation into the mechanisms of MDR in ATC. Consistent with these findings, we observed that NSUN2 knockout significantly inhibited the N-linked glycosylation of ABCC1 and ABCG2, promoting ubiquitin-mediated degradation and subsequently enhancing ATC sensitivity to doxorubicin, cisplatin, and lenvatinib. To enhance the clinical relevance of this research, we synthesized a previously described NSUN2 inhibitor 35. We employed a genetically engineered mouse model of ATC to evaluate the *in vivo* efficacy of the NSUN2 inhibitor in combination with chemotherapy agents or TKIs. The results demonstrated that the NSUN2 inhibitor markedly enhanced the antitumor effects of doxorubicin, cisplatin, and lenvatinib, suggesting that NSUN2 is a promising target for overcoming multidrug resistance in ATC.

Although our promising results underscore the potential of NSUN2 inhibitor in combination with chemotherapy or TKIs for the treatment of ATC, it is important to note that NSUN2 plays fundamental roles in normal cellular physiology, and systemic NSUN2 inhibition may pose safety concerns. Therefore, comprehensive preclinical studies are essential to thoroughly evaluate the toxicological profile of NSUN2 inhibitor, including dose-limiting toxicities, acute and chronic toxicity, as well as genotoxicity and carcinogenicity. Additionally, effective tumor-specific delivery of NSUN2 inhibitor remains a significant challenge. Consequently, exploring advanced drug delivery systems, such as nanoparticle-based carriers and ligand-targeted approaches, is of great value to enhance tumor-specific accumulation and reduce potential toxicity to normal tissues.

## Conclusions

In summary, our work on the NSUN2/SRSF6/UAP1 signaling axis offers novel insights into m^5^C modification and alternative splicing, while providing potential therapeutic targets for improving chemotherapy and targeted therapy outcomes in ATC.

## Supplementary Material

Supplementary figures and tables.

## Abbreviations

ATC: Anaplastic thyroid carcinoma
mATC: Murine anaplastic thyroid carcinoma
mNT: Murine normal thyroid tissue
MDR: Multidrug resistance
AS: Alternative splicing
NSUN2: NOP2/Sun RNA methyltransferase family member 2
SRSF6: Serine and arginine rich splicing factor 6
UAP1: UDP-N-Acetylglucosamine pyrophosphorylase 1
AGX1: Asparagine-linked glycosylation 1
AGX2: Asparagine-linked glycosylation 2
CTRP: The Cancer Therapeutics Response Portal
ABCG2: ATP-binding cassette subfamily G member 2
ABCC1: ATP-binding cassette subfamily C member 1
TKIs: Tyrosine kinase inhibitors
ALYREF: Aly/REF export factor
MeRIP-seq: Methylation-RNA immunoprecipitation sequencing
MFI: Mean fluorescence intensity
