# Supplementary material for: NSUN2-mediated m5C modification drives alternative splicing reprogramming and promotes multidrug resistance in anaplastic thyroid cancer through the NSUN2/SRSF6/UAP1 signaling axis
Source: Theranostics. 2025 Jan 27;15(7):2757–77. doi: 10.7150/thno.104713 (PMC11898302; doi:10.7150/thno.104713)
Supplement: Supplementary file 1 — Supplementary figures and tables. [file thnov15p2757s1.pdf]

NSUN2-mediated m^{5}C modification drives alternative splicing reprogramming and promotes multidrug resistance in anaplastic thyroid cancer through the NSUN2/SRSF6/UAP1 signaling axis

Xiukun Hou, Qiman Dong, Jie Hao, Min Liu, Junya Ning, Mei Tao, Zhongyu Wang, Fengli Guo, Dongmei Huang, Xianle Shi, Dapeng Li, Ming Gao, and Xiangqian Zheng

Figure S1

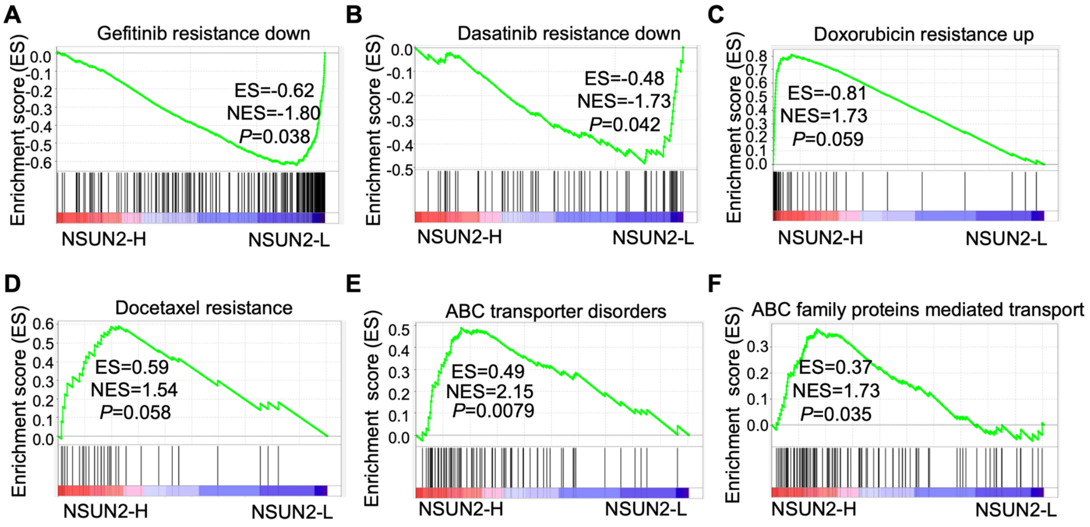

Bioinformatics analysis reveals a correlation between NSUN2 and MDR in ATC

A-D) GSEA enrichment analysis shows that NSUN2 is correlated with resistance to chemotherapy drugs and TKIs. E-F) GSEA enrichment analysis shows that NSUN2 is correlated with indicated cellular processes.

Figure S2

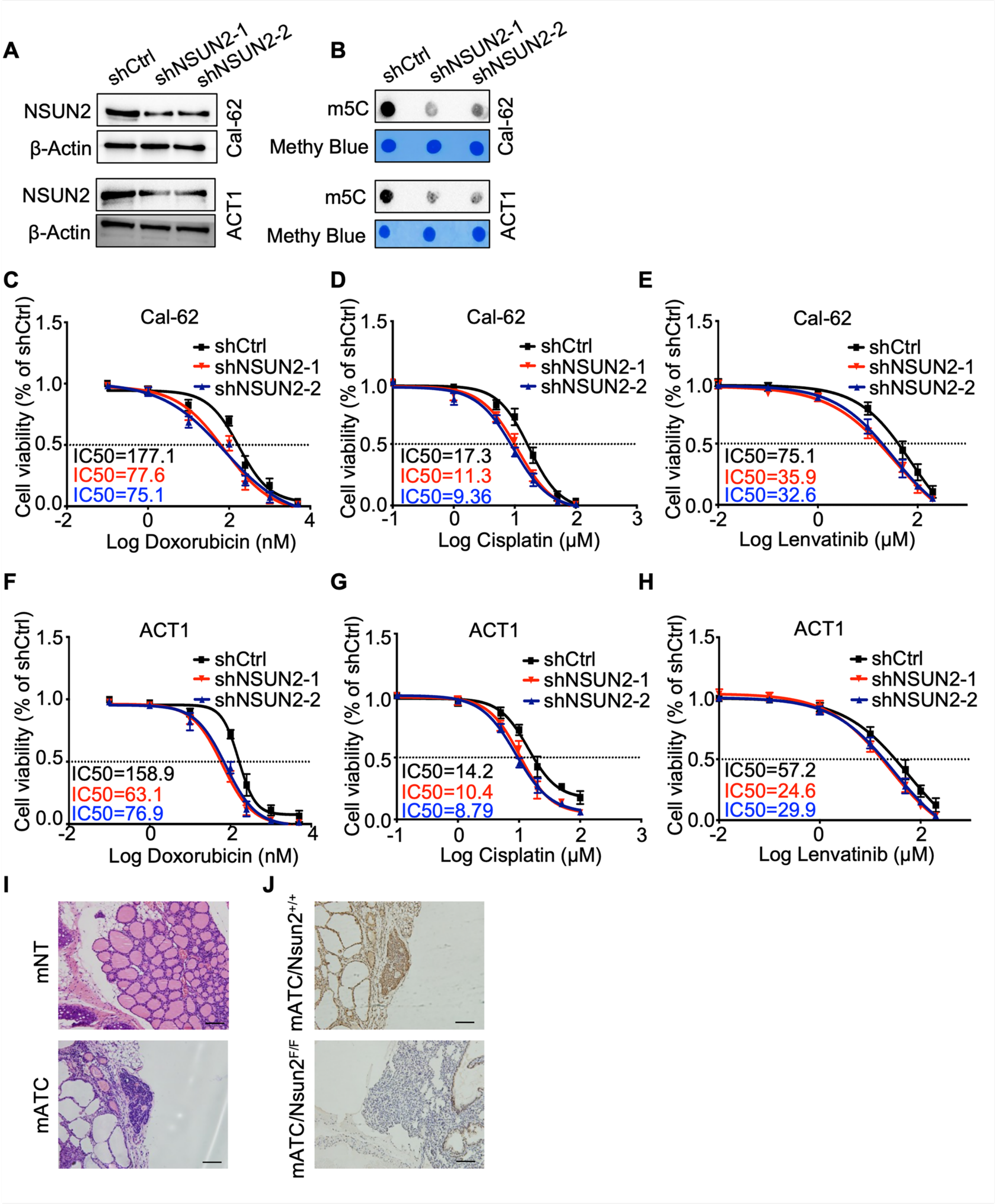

NSUN2-induced MDR in ATC depends on its methyltransferase activity

A) Western blot analysis of NSUN2 protein levels in ATC cells with NSUN2 knockdown. B) Effect of NSUN2 knockdown on m^5C abundance in mRNA transcriptomes of ATC cells, assessed by dot blot assay. C-H) Cell growth inhibition assay evaluating the impact of NSUN2 knockdown on ATC cells response to doxorubicin, cisplatin, and lenvatinib. I) Representative H&E staining images of

the thyroid gland in genetically engineered mice. J) Representative IHC staining images of the thyroid gland in genetically engineered mice. mATC (murine anaplastic thyroid carcinoma), mNT (murine normal thyroid tissue). The data are presented as the mean ± SD. All $*p < 0.05$, $**p < 0.01$, $***p < 0.001$.

Figure S3

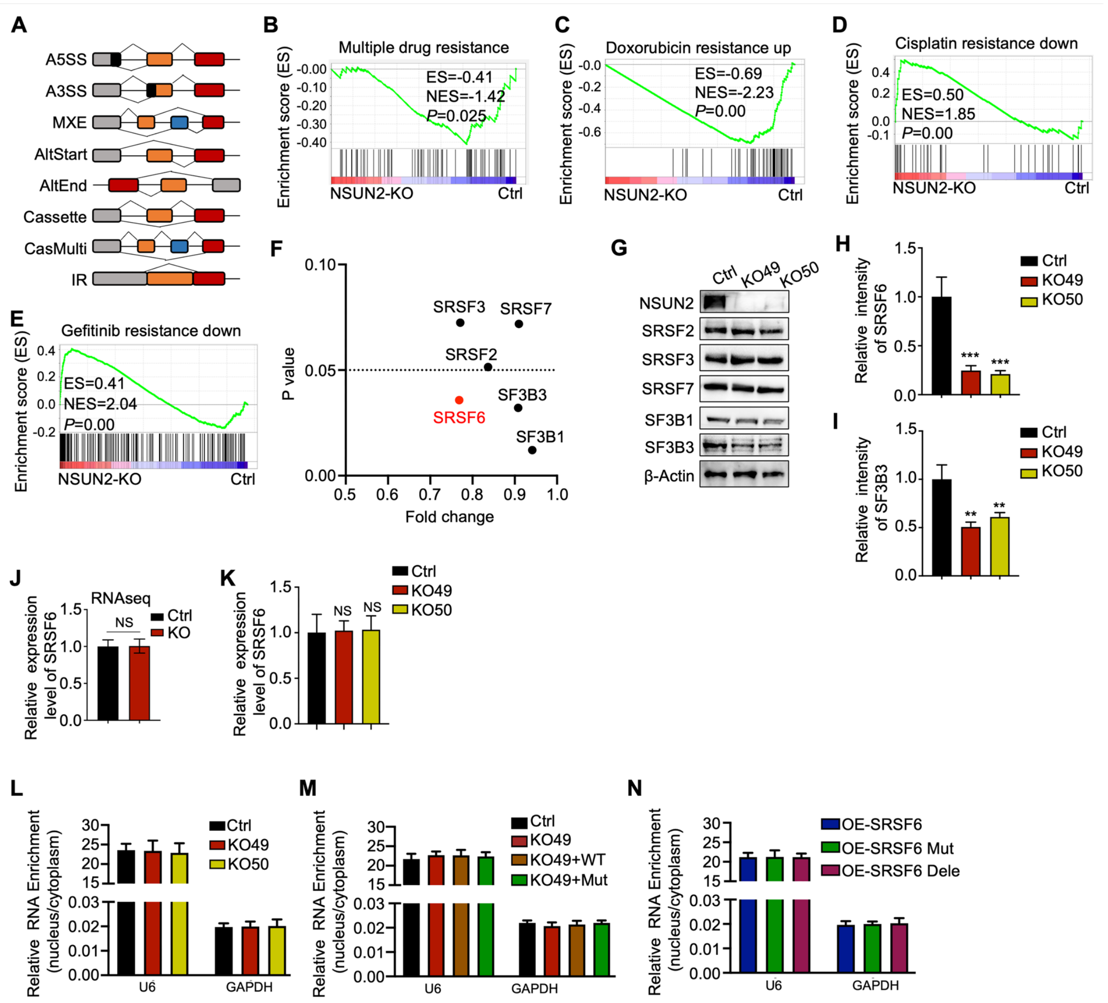

NSUN2-mediated m^{5}C modification drives alternative splicing reprogramming by targeting SRSF6 in ATC

A) Schematics of the eight types of AS events. A5SS: alternative 5´splice site; A3SS: alternative 3´splice site; MXE: mutually exclusive exon; AltStart: alternative start exon; AltEnd: alternative end exon; Cassette: cassette exon; CasMulti: multiple adjacent cassette; IR: intron retention. B-E) GSEA enrichment analysis of RNA-seq data in Cal-62 cells upon NSUN2 knockout. F) Volcano plot of the indicated genes from TMT-MS data. G) Western blot of indicated protein levels in Cal-62 cells knocking out NSUN2. H-I) Statistical quantification of SRSF6 and SF3B3 expression levels according to the western blot from panel G. J) Statistical quantification of SRSF6 expression level

according to the RNA-seq data. K) RT-qPCR of SRSF6 level in Cal-62 cells knocking out NSUN2. L-N) Nuclear and cytoplasmic distribution of U6 and GAPDH mRNA in Cal-62 cells treated with indicated vectors. The data are presented as the mean ± SD. All $*p < 0.05$, $**p < 0.01$, $***p < 0.001$.

Figure S4

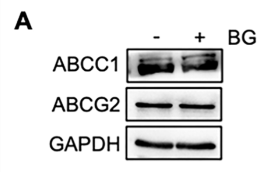

NSUN2 promotes MDR in ATC by enhancing the N-glycosylation of ABC transporters

A) Western blotting analysis of indicated protein levels in Cal-62 cells treated with or without Benzyl-α-N-acetylgalactosamine (BAG).

Figure S5

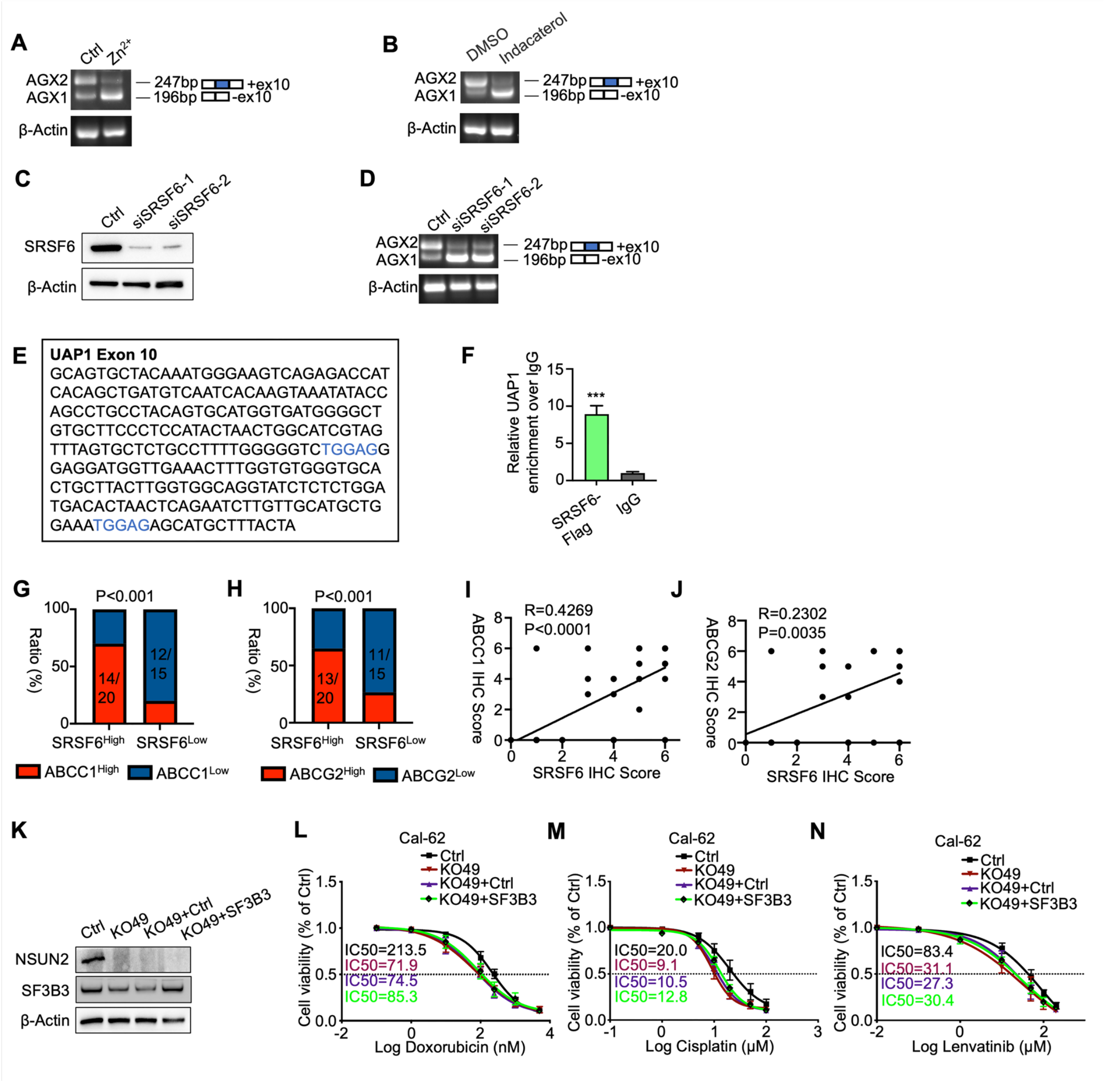

NSUN2 promotes N-glycosylation by mediating the alternative splicing of UAP1 through SRSF6

A-B) RT-PCR showing the splicing events of UAP1 in Cal-62 cells treated with zinc ions (Zn^{2+}) or indacaterol. The number of skipped exons is depicted for each transcript. C) Western blotting analysis of SRSF6 level in Cal-62 cells treated with small interfering RNA. D) RT-PCR showing the splicing events of UAP1 in Cal-62 cells treated with small interfering RNA. The number of skipped exons is depicted for each transcript. E) The consensus motif sequence (TGGAG) with predicted SRSF6 binding motif in exon10 of UAP1. F) RIP-qPCR analysis of SRSF6 binding ability

to UAP1 in Cal-62 cells expressing SRSF6. G-H) The chi-square test was used to analyze the correlation between SRSF6 and ABCG2, ABCC1 expression level. I-J) The correlation analysis of IHC scores between SRSF6 and ABCG2, ABCC1. K) Western blotting analysis of SF3B3 level in Cal-62 cells treated with indicated vectors. L-N) Cell growth inhibition assays evaluating the effects of the indicated vectors on Cal-62 cell sensitivity to doxorubicin, cisplatin, and lenvatinib. The data are presented as the mean ± SD. All $^{*}p < 0.05$, $^{**}p < 0.01$, $^{***}p < 0.001$.

Figure S6

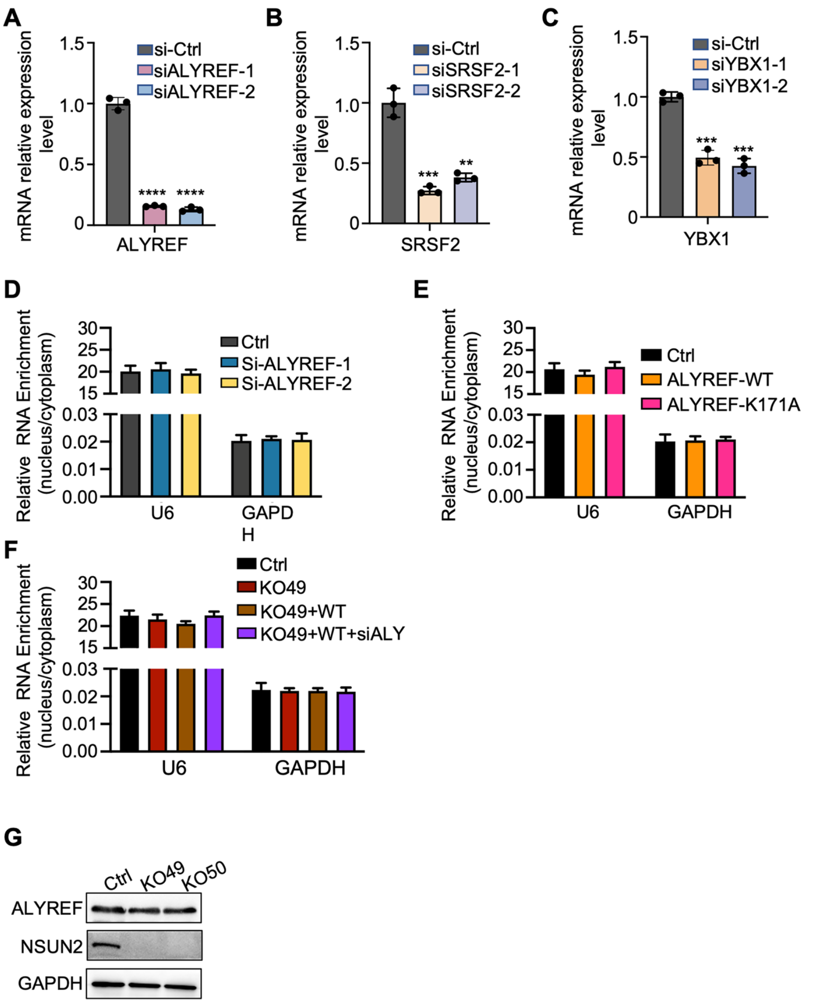

ALYREF functions as an m^{5}C "reader" that recognizes and shuttles SRSF6 from nucleus to cytoplasm

A-C) The knockdown efficiencies of ALYREF, YBX2, and SRSF2 were evaluated by qRT-PCR assay. D-F) Nuclear and cytoplasmic distribution of U6 and GAPDH mRNA in Cal-62 cells treated with indicated small interfering RNAs or vectors. G) Western blotting analysis of indicated proteins level in Cal-62 cells with NSUN2 knockout. The data are presented as the mean ± SD. All *$p < 0.05$, **$p < 0.01$, ***$p < 0.001$.

Figure S7

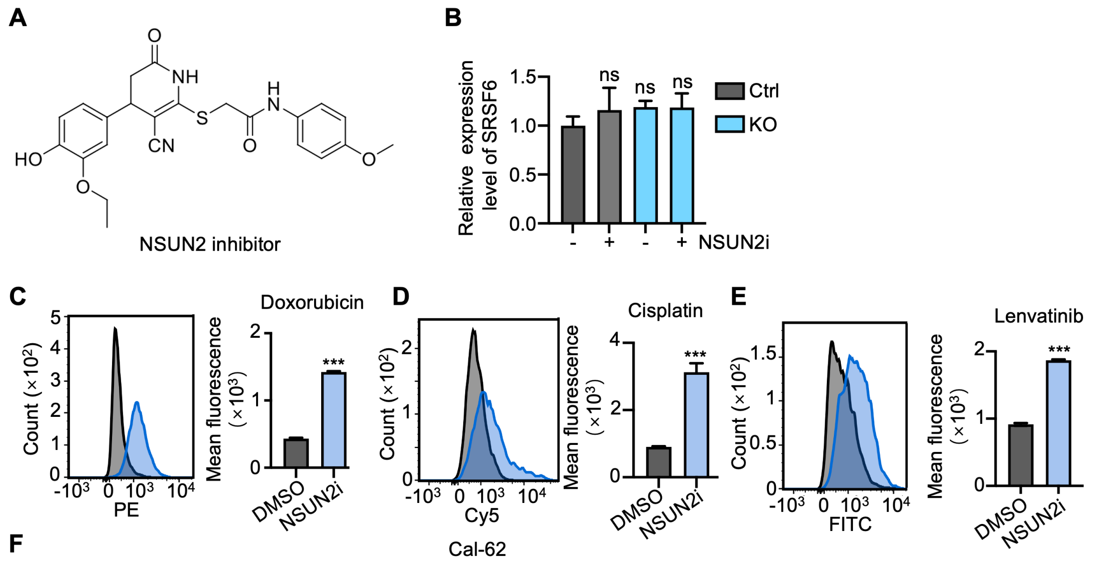

F

Cal-62

Doxo (nM)	NSUN2i (μM)	Combination Index (CI)	Cisp (μM)	NSUN2i (μM)	Combination Index (CI)	Lenv (μM)	NSUN2i (μM)	Combination Index (CI)
25	15	0.952	4	15	0.836	10	15	0.719
50	15	0.735	8	15	0.764	20	15	0.697
100	15	0.260	16	15	0.602	40	15	0.396
25	30	0.847	4	30	0.854	10	30	0.724
50	30	0.578	8	30	0.633	20	30	0.408
100	30	0.242	16	30	0.380	40	30	0.314

G

ACT-1

Doxo (nM)	NSUN2i (μM)	Combination Index (CI)	Cisp (μM)	NSUN2i (μM)	Combination Index (CI)	Lenv (μM)	NSUN2i (μM)	Combination Index (CI)
25	15	0.855	4	15	0.864	10	15	0.801
50	15	0.514	8	15	0.554	20	15	0.619
100	15	0.344	16	15	0.451	40	15	0.365
25	30	0.814	4	30	0.805	10	30	0.781
50	30	0.501	8	30	0.578	20	30	0.447
100	30	0.276	16	30	0.366	40	30	0.236

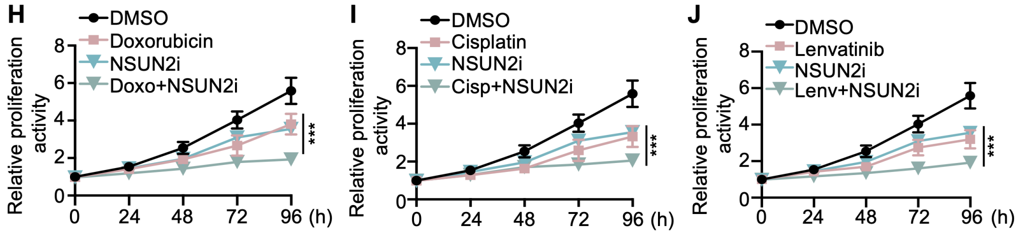

Small-molecule NSUN2 inhibitor enhances multidrug sensitivity in ATC

A) 2D structure of small-molecule inhibitor of NSUN2, which labeled with Nsun2-i4 in the original research [1]. B) RT-qPCR showing the SRSF6 mRNA level in Cal-62 cells knocking out NSUN2 combined with or without NSUN2 inhibitor. C-E) Flow cytometry analysis of intracellular

accumulation of doxorubicin, cisplatin, and lenvatinib in ACT-1 cells treated with or without NSUN2 inhibitor (left). Quantification of mean fluorescence intensity was shown (right). F-G) The combination index (CI) values of NSUN2 inhibitor combined with doxorubicin, cisplatin, or lenvatinib in Cal-62 cells and ACT-1 cells. H-J) Cell growth inhibitory assay to evaluate the impacts of ACT-1 cells treated with NSUN2 inhibitor combined with doxorubicin, cisplatin, or lenvatinib. The data are presented as the mean ± SD. All $*p < 0.05$, $**p < 0.01$, $***p < 0.001$.

Table S1. primers used for cloning and siRNA sequences used in this study

Target Gene	Sequence 5′→ 3′
sgNSUN2	GAGCTCAAGATCGTGCCCGA
shNSUN2-2	TGCAGTGTCCCATCGTCTTAT
shNSUN2-3	TGAGAAGATGAAGGTTATTAA
OE-NSUN2	F: GATGACGATGACAAGCTCGAGatggggcggcggtcgcggggt<br>R: GCGGCCGCTCTAGAACTAGTtcaccggggtggatggacccc
OE-NSUN2 C271A Mut	F: atgtccctGCcagtggagacggcactat<br>R: gtctccactgGCagggacatcacataaaattcg
OE-SRSF6	F: ACCTCCATAGAAGATTCTAGAGCCACCATGCCGCGCGTCTACATA<br>R: TGGTCTTTGTAGTCGGATCCATCTCTGGAACTCGACCTG
OE-ABCC1	F: atggcgctccggggcttctgcagc<br>R: tcacaccaagccggcgtctttggc
OE-ABCG2	F: CTCTCCAGATGTCTTCCAGT<br>R: ACAGTGTGATGGCAAGGGAAC
OE-ALYREF	F: GATTCTAGAGCTAGCGAATTCGCCACCatgcccgattccgcgcccgc<br>R: ATGGTCTTTGTAGTCGGATCCactggtgtccattctcgc
OE-ALYREF K171A Mut	F: ctgaaggccatgGCTcagtacaacggcgtccct<br>R: tactgAGCcatggccttcagggcatc
siALYREF-1	sense: GCGAGAAUGGACACCAGUUTT

	antisense: AACUGGUGUCCAUUCUCGCTT
siALYREF-2	sense: UGGGAAACUGCUGGUGUCCAATT<br>antisense: UUGGACACCAGCAGUUUCCCATT
siYBX1-1	sense: CAGUUCAAGGCAGUAAAUAUGCA<br>antisense: UGCAUAUUUACUGCCUUGAACUG
siYBX1-2	sense: CGGCAAUGAAGAAGAUAAAdTdT<br>antisense: UUUAUCUUCUUCAUUGCCGdTdT
siSRSF2-1	sense: GCACGAAGGUCCAAGUCCATT<br>antisense: UGGACUUGGACCUUCGUGCTT
siSRSF2-2	sense: CCGAUUGCUCCUGUGUAAATT<br>antisense: UUUACACAGGAGCAAUCGGTT
siSRSF6-1	sense: GCGUCUACAUAGGACGCCUGAGCUA<br>antisense: UAGCUCAGGCGUCCUAUGUAGACGC
siSRSF6-2	sense: CCUGUUCGUACAGAAUACAGGCUUA<br>antisense: UAAGCCUGUAUUCUGUACGAACAGG

Table S2. The antibodies used in this study.

Antibody	Supplier	Catalogue	Host
Anti-NSUN2	Proteintech	20854-1-AP	Rabbit
Anti-SRSF6	ABclonal	A14603	Rabbit
Anti-m^5C	Abcam	ab214727	Rabbit

Anti-ABCC1	Proteintech	67228-1-Ig	Mouse
Anti-ABCG2	Proteintech	27286-1-AP	Rabbit
Anti-ALYREF	Abcam	ab202894	Rabbit
Anti-ALYREF	Proteintech	16690-1-AP	Rabbit
Anti-PARP1	CST	9542	Rabbit
Anti-B-ACTIN	BOSTER	BM0627	Mouse
Anti-UAP1	Proteintech	67545-1-Ig	Mouse
Anti-Ubiquitin	Proteintech	10201-2-AP	Rabbit

Table S3. The small-molecule compounds or drugs used in our study

Name	Supplier	Catalogue
Doxorubicin	MCE	HY-15142
Cisplatin	GLPBIO	GC11908
Lenvatinib	GLPBIO	GC15454
PNGase F	NEB	P0704S
MG132	APExBIO	A2585
Cycloheximide	APExBIO	A8244
Benzyl-α-N-acetylgalactosamine	MCE	HY-129389
Tunicamycin	APExBIO	B7417
Swainsonine	APExBIO	B7316

Table S4. Primers for qRT-PCR assay

Gene	Forward primer 5´→ 3´	Reverse primer 5´→ 3´
SRSF6	CCAATTCGCCGCTACCTGTTC	CCTGGACCTTGATCTTGACTTTGAG
GAPDH	GAAGGTGAAGGTCGGAGTC	GAAGATGGTGATGGGATTTC
U6	CTCGCTTCGGCAGCACA	AACGCTTCACGAATTTGCGT
ALYREF	TGGCTTCGGCGGTGGTG	GTTCCTGAATATCGGCGTCTGAG
YBX1	AGGAGAACAAGGTAGACCAGTGAG	AGGCTGTCTTTGGCGAGGAG
SRSF2	ACGCTGAGGACGCTATGGATG	CGGCGGCTGTGGTGTGAG

Table S5. Primers for RIP-qPCR assay

Gene	Forward primer 5´→ 3´	Reverse primer 5´→ 3´
SRSF6	ACATAGGACGCCTGAGCTACA	GCCGTACCCATTTTTGAGGTCTA
UAP1	TGCTTCCATTCCCATTAATCCT	CTGTAGGCAGGCTGGTATATTT

Table S6. Primers for RT-PCR assay

Gene	Forward primer 5´→ 3´	Reverse primer 5´→ 3´
UAP1	GCTGATAGTCAGAATGGGAAAGA	CAGCATAGGAGATAAGAGGAGAGA

Table S7. Mouse genotypes primers

Gene	Forward primer 5´→ 3´	Reverse primer 5´→ 3´
$BRAF^{V600E}$	TGAGTATTTTTGTGGCAACTGC	CTCTGCTGGGAAAGCGGC
P53	CACAAAAAACAGGTTAAACCCAG	AGCACATAGGAGGCAGAGAC

	GAAGACAGAAAAGGGGAGGG	
TPO	CTAGCATCTTGACGGGCTATC	CTAGGCCACAGAATTGAAAGATCT
	GTAGGTGGAAATTCTAGCATCATCC	AGGCAAATTTTGGTGTACGG
NSUN2	GAGTCACATCAAAGCCCTTGTTC	AGCTATGCAGACTGAAGAATGAAG

Reference

1. Chen B, Deng Y, Hong Y, Fan L, Zhai X, Hu H, et al. Metabolic Recoding of NSUN2-Mediated m(5)C Modification Promotes the Progression of Colorectal Cancer via the NSUN2/YBX1/m(5)C-ENO1 Positive Feedback Loop. Adv Sci. 2024; 11: e2309840.
